# Preclinical evaluation of FLT190, a liver-directed AAV gene therapy for Fabry disease

**DOI:** 10.1038/s41434-022-00381-y

**Published:** 2023-01-11

**Authors:** Jey M. Jeyakumar, Azadeh Kia, Lawrence C. S. Tam, Jenny McIntosh, Justyna Spiewak, Kevin Mills, Wendy Heywood, Elisa Chisari, Noemi Castaldo, Daniël Verhoef, Paniz Hosseini, Petya Kalcheva, Clement Cocita, Carlos J. Miranda, Miriam Canavese, Jaminder Khinder, Cecilia Rosales, Derralynn Hughes, Rose Sheridan, Romuald Corbau, Amit Nathwani

**Affiliations:** 1 Freeline Therapeutics, Ltd, Stevenage, UK; 2 University College London, London, UK; 3 UCL Biological Mass Spectrometry Centre, Genetics & Genomic Medicine Unit, Institute of Child Health, UCL, London, UK; 4 Royal Free London NHS Foundation Trust, London, UK

**Keywords:** Metabolic disorders, Diseases

## Abstract

Fabry disease is an X-linked lysosomal storage disorder caused by loss of alpha-galactosidase A (α-Gal A) activity and is characterized by progressive accumulation of glycosphingolipids in multiple cells and tissues. FLT190, an investigational gene therapy, is currently being evaluated in a Phase 1/2 clinical trial in patients with Fabry disease (NCT04040049). FLT190 consists of a potent, synthetic capsid (AAVS3) containing an expression cassette with a codon-optimized human *GLA* cDNA under the control of a liver-specific promoter FRE1 (AAV2/S3-FRE1-GLAco). For mouse studies FLT190 genome was pseudotyped with AAV8 for efficient transduction. Preclinical studies in a murine model of Fabry disease (*Gla*-deficient mice), and non-human primates (NHPs) showed dose-dependent increases in plasma α-Gal A with steady-state observed 2 weeks following a single intravenous dose. In Fabry mice, AAV8-FLT190 treatment resulted in clearance of globotriaosylceramide (Gb3) and globotriaosylsphingosine (lyso-Gb3) in plasma, urine, kidney, and heart; electron microscopy analyses confirmed reductions in storage inclusion bodies in kidney and heart. In NHPs, α-Gal A expression was consistent with the levels of h*GLA* mRNA in liver, and no FLT190-related toxicities or adverse events were observed. Taken together, these studies demonstrate preclinical proof-of-concept of liver-directed gene therapy with FLT190 for the treatment of Fabry disease.

## Introduction

Fabry disease is a rare, X-linked lysosomal storage disease that is caused by a mutation in the gene encoding the enzyme alpha-galactosidase A (α-Gal A) [1]. A marked reduction or absence of α-Gal A activity results in progressive accumulation of substrates globotriaosylceramide (Gb3), globotriaosylsphingosine (lyso-Gb3) and related glycosphingolipids in multiple cell types resulting in a multi-system disorder affecting both males and females. Current standard of care for the treatment of Fabry disease consists of adjunctive therapies for management of symptoms and complications, and chronic/life-long disease-modifying therapies aimed at increasing the availability of functional lysosomal α-Gal A. Approved disease-modifying therapies for Fabry include enzyme replacement therapy (ERT) with intravenous recombinant or gene-activated α-Gal A (agalsidase-alfa or agalsidase-beta) and oral pharmacological chaperone therapy (PCT) with migalastat [2]. Treatment with ERT has been shown to slow disease progression [3, 4], however, the efficacy of ERT is limited and variable with most patients still experiencing cardiac, renal, and cerebral complications [2], immune intolerance, infusion-related discomfort, and the formation of neutralizing antibodies against ERT. Furthermore, ERT treatment imposes a life-long burden of biweekly intravenous infusions. Migalastat selectively and reversibly binds to the active site of α-Gal A, thereby correcting the misfolding of the enzyme and allowing it to traffic to the lysosome [5]. While migalastat has a similar efficacy profile as ERT, it can only be used by ~35–50% of patients with Fabry disease who have amenable *GLA* gene variants [6]. Hence, there is an unmet need for more effective and convenient treatments for Fabry disease.

Adeno-associated virus (AAV) gene therapy has progressed rapidly over the past decade with the advent of novel capsid serotypes, tissue-specific promoters, and an increased understanding of the immune response to AAV [7, 8]. A liver-directed AAV-mediated gene transfer approach may be effective for the treatment of Fabry disease by transducing hepatocytes with a functional copy of the *GLA* gene, leading to continuous, endogenous expression of α-Gal A protein, which is then transported to Fabry-affected tissues via the blood. AAV gene therapy has the potential to provide continuous production of functional α-Gal A enzyme, which would eliminate the brief peaks and prolonged troughs in plasma α-Gal A levels observed with ERT. Furthermore, it is hypothesized that patient-specific post-translational modifications of the endogenously expressed α-Gal A protein following AAV gene therapy may result in greater penetration into key tissues (kidney and heart) than with ERTs [9] which may lead to improved clearance of glycosphingolipids and improved efficacy. It is also recognized that liver-directed AAV gene therapies may induce immune tolerance to transgene products [10], thereby overcoming the anti-drug antibody formation associated with ERT in Fabry patients.

The potential of using rAAV gene therapy for Fabry disease has been demonstrated in a mouse model of Fabry by several groups [11–16], the first of which was reported by Jung et al. in 2001 [11] using the human EF1-a promoter. Several follow-up studies have demonstrated sustained production of α-Gal A in the Fabry mouse model and non-human primates (NHPs) following a single intravenous infusion of recombinant AAV8 vector with a liver-specific promoter [15]; however, these studies suggest that relatively large vector doses would be required to achieve therapeutic levels in humans [14, 15].

FLT190 is an investigational AAV gene therapy in development as a potential treatment for Fabry disease and is currently being studied in a Phase 1/2 clinical trial (NCT04040049). FLT190 consists of a potent, rationally designed capsid (AAVS3) containing an expression cassette with a codon-optimized human *GLA* cDNA under the control of a proprietary liver-specific promoter (FRE1). The AAVS3 capsid was constructed by rational design from carefully selected domains of natural AAV serotypes, and preferentially targets the human liver allowing for functional transduction and subsequent protein production at therapeutic levels. We have demonstrated the superior transduction efficiency of our AAVS3 capsid in primary human hepatocyte cell cultures compared with AAV5 and AAV8 variants currently used in the clinic [17]. We have also confirmed this in a humanized mouse liver model, as well as in rhesus macaques dosed with AAVS3-pseudotyped factor IX vectors [17]. Here we report the characterization of our investigational AAV gene therapy, FLT190, in in vitro and in vivo models.

## Materials and methods

### In vitro studies

To demonstrate α-Gal A uptake in multiple wild type and enzyme-deficient cell lines (hepatocytes, cardiomyocytes, kidney epithelial, podocytes, patient-derived Fabry fibroblasts and microvascular derived endothelial cells), the level of enzyme uptake into representative cell lines of Fabry disease was evaluated using the Transwell co-culturing system with apical and basal compartments (Supplementary Methods). In all experiments (as indicated otherwise), human liver cells (Huh7) were seeded in apical transwells and subjected to AAV transduction, and cells co-cultured in basal transwells were exposed to secreted enzyme for uptake. In some experiments, a stable Huh7 cell line overexpressing α-Gal A (*GLA*co) from an integrated lentiviral vector was used. α-Gal A uptake into co-cultured cells were then evaluated by A) Western blot analysis for total cellular α-Gal A protein (Supplementary Methods), B) cellular α-Gal A enzyme activity, and C) confocal microscopic examination of internalized α-Gal A colocalizing with lysosomal markers.

### Recombinant AAV vectors

AAV expression cassettes were generated by cloning the cDNA of a codon-optimized human *GLA* under the control of the liver-specific promoter FRE1 into single-stranded AAV backbone plasmids. An additional *GLA* construct with an HA-tag before the stop codon of *GLA* was also generated by Q5-site directed mutagenesis. HA tag allows detection of α-Gal A derived from FLT190 transduction of Huh7 cells versus endogenous protein.

For AAV virus production, relevant batches of viral particles (AAV8 or AAVS3) were produced by triple transient transfection of HEK293T cells with plasmids encoding the AAV Rep and Cap functions (AAV2/8: rep2-AAV8 or AAV2/S3: pREP2-S3), adenoviral helper functions (HGTI), and the recombinant genome containing *GLA* expression cassette flanked by AAV2 ITRs. AAV vectors were purified by affinity chromatography using POROS™ CaptureSelect™ AAV8 Affinity Resin (Life Technology) or AVB Sepharose (for AAVS3). Purified stocks were titered by qPCR and characterized by alkaline gel analysis.

### Cell culture and AAV transduction

All cell lines were maintained at 37 ^o^C with 5% CO_2_ unless otherwise stated. Huh7 cells were obtained from the Japanese Collection of Research Bioresources (JCRB) cell bank and maintained in D-10 media (DMEM, 10% FBS, 1% L-glutamax). Human kidney epithelial cells (HK2) were maintained in K-SFM media supplemented with BPE and EGF. Human cardiomyocytes (AC16) were maintained in DMEM/F-12 media supplemented with 1X L-glutamax, 12.5% FBS. Human podocytes were maintained in RPMI media supplemented with 1X ITS and 10% FBS. Fabry patient-derived microvascular endothelial cells (IMFE1) were provided by Dr. Jinsong Shen (NINDS, NIH, US) [18], and maintained in endothelial cell growth media (R&D Systems). Fabry patient and healthy human-derived fibroblasts were obtained from Coriell Institute and maintained in D-10 media (DMEM, 10% FBS, 1% L-glutamax). A human podocyte cell line (AB 8/13) was provided by Prof. Moin A. Saleem (University of Bristol, Bristol, UK [19]) and maintained in RPMI-1640 supplemented with Insulin-Transferrin-Selenium and 10% FBS. AB 8/13 was proliferated at 33 ^o^C (in 5% CO_2_) until confluency was reached, and then induction of differentiation was performed by incubating the cells at 37 ^o^C (in 5% CO_2_) for 2 weeks before experimentation.

For AAV transduction, appropriate cell numbers were seeded in multi-well plates (2 × 10^5^ or 2 × 10^4^ cells/well, 12-well or 96-well plate respectively), and the required AAV multiplicity of infection (MOI) was added in X-VIVO 10 media (Lonza) containing L-glutamine. The cells were allowed to adhere for 24 h before transduction. Following the 24 h incubation, the transduction mix of D10 containing the appropriate titer of AAV viral particles for the particular experiment was prepared and added to the cells (500 μL or 50 μL/well for 12-well or 96-well plate respectively); plates were then incubated overnight at 37 °C, 5% CO_2_. The following day, the transduction mix was removed by aspiration, and new D-10 media was added to the wells. A further 48 h later, the media and cells were collected and analyzed for determination of α-Gal A activity.

For human primary hepatocytes (Lot No HUM1981, Lonza), cells were seeded in collagen-coated 96-well plates and transduced with FLT190 vectors at different MOI. Twenty-four hours post-transduction, cell culture media was replaced with fresh media and incubated for an additional 48 h before analysis. Details on the generation of knockdown and stable *GLA* Huh7 cell lines are provided in Supplementary Methods.

### Gb3 analysis

Quantitation of Gb3 and Lyso-Gb3 levels were determined by tandem mass spectrometry (LC-MS/MS) as describe previously [20, 21].

For tissue samples, a small amount of wet tissue 10–50 mg was homogenized using a ceramic bead homogenizer in ammonium bicarbonate buffer. Homogenate was spun and the supernatant assayed for protein content. Five hundred micrograms of liver homogenate were freeze dried and reconstituted in phosphate buffered saline. Extraction solution containing an internal standard was added to the homogenate tissue and then shaken and sonicated. The solution was spun and lipid containing solution was removed and concentrated prior to being re-suspended and analysis by LC-MS/MS [20].

For plasma and urine samples, samples volumes (10 to 100 microliter) were collected at room temperature and stored at −80 °C until analysis by mass spectrometry. Glycosphingolipids were extracted as previously described [20].

### Determination of vector genome copy number

Frozen tissue samples (weighing ~ 50 mg) were processed for DNA extraction. Samples were homogenized in Precellys 2 mL CK-mix homogenizing tubes (Bertin Instruments, cat# P000918-LYSK0-A) containing 360 µl of ATL buffer (provided by Qiagen DNeasy Blood & Tissue Kit, cat #/ID: 69506) using a Precellys Evolution Tissue Homogenizer (Bertin Instruments; 3 × 20 s homogenization, 30 s break, 6800 rpm). Tissue lysates were digested with 35 µL of Proteinase K (Qiagen DNeasy Blood & Tissue Kit; 56 °C with agitation at 550 rpm for 2 h), treated with 6 µL of RNAse A (100 mg/mL) mixed and incubated for 2 min at RT. DNA purification was carried out using the Qiagen DNeasy Blood & Tissue kit following manufacturer’s instructions. DNA content was quantified using NanoDrop spectrophotometer (NanoDrop™ 2000 Spectrophotometer, Thermo Scientific™) and DNA samples at 10 ng/µL were used for qPCR analysis. Viral genome copy number was determined using primers that bind specifically to FRE1 promoter sequence (FW: 5’-TTGCTCCTCCGATAACTG-3’, REV: 5’-GTGCCTGAAGCTGAGGAGAC-3’). Quantitation of the viral genome was achieved using a dilution curve (1 × 10^8^ to 1 × 10^3^ vg per reaction) using a linearized plasmid prepared for Freeline by Aldevron. PCR was performed using a QuantStudio Real-Time qPCR machine (ThermoFisher) using the standard cycling mode for primers with a Tm ≥60 °C. Analysis was performed over a total of 40 cycles. qPCR data was analyzed with QuantStudio Design and Analysis Software. The number of viral genomes present in a sample were calculated by reference to the relevant standard curve and were expressed as viral copies per cell.

### In vivo studies

Animal experiments were performed in accordance with the provisions of the United Kingdom Animals (Scientific Procedures) Act 1986 Amendment Regulations 2012 (the Act). All study mice were housed in the barrier facilities with an automatic watering system and husbandry by Veterinary care under UK Home Office regulations and in accordance with Animal Welfare regulations at Royal Free Hospital, London, UK, where the studies were conducted. The study protocols were used under Freeline UK Home Office Project Licence.

### Wild-type (WT) mice

FLT190 genome (FRE1-GLAco) pseudotyped with an rAAV8 vector (AAV8-FLT190) was used for mouse studies. AAV8-FLT190 was administered into the tail vein of wild-type C57BL6 mice at 2 months of age. The following three dose groups were studied: AAV8-FLT190 6 × 10^11^ vg/kg (*n* = 5), AAV8-FLT190 2 × 10^12^ vg/kg (*n* = 8), and AAV8-FLT190 6 × 10^12^ vg/kg (*n* = 4), and an additional group left as untreated to serve as a control (*n* = 4). To assess the kinetics and durability of transgene expression, plasma α-Gal A levels were measured at 2, 4, 8, and 11 weeks post-injection. After the 11-week time point, mice were sacrificed for biochemical analysis.

For ERT studies, agalsidase alfa (Replagal®, Shire Human Genetic Therapies Inc., Cambridge, MA, USA) and agalsidase beta (Fabrazyme®, Genzyme, Cambridge, MA, USA) were reconstituted according to package instructions to give nominal protein concentrations of 1 mg/mL and 5 mg/mL, respectively. Agalsidase alfa (0.2 mg/kg) or agalsidase beta (1 mg/kg) was administrated into the tail vein of C57BL6 wild-type mice at 2 months of age (*n* = 15 per group). Mice were sacrificed at 10 min (*n* = 5), 24 h (*n* = 5) or 1 week (*n* = 5) post-injection for determination of α-Gal A activity levels in plasma. ERT commercial products were purchased via Royal Free London NHS Foundation Trust (Royal Free Hospital Pharmacy Department) for research purposes.

### Gla-deficient (Fabry) mice

Fabry mice carrying a targeted disruption of the α-Gal A gene (*Gla*) (C57BLy6; *Gla*-deficient mice) have been described previously [22]. A live colony was maintained by breeding homozygous females to hemizygous males (The Jackson Laboratory) B6;129-Gla^tm/Kul^ /J Stock No: 003535 | α-Gal A KO). AAV8-FLT190 was administered into the tail vein of *Gla*-deficient mice [22] at 4 months of age. One group of *Gla*-deficient mice received AAV8-FLT190 2 × 10^12^ vg/kg (5 male and 6 female), and a second group left as untreated to serve as a control for the effects of treatment (3 male and 2 female); a third group of untreated wild-type mice (4 male) served as a control group. To assess the kinetics and durability of transgene expression, plasma α-Gal A levels were measured at 2, 4, 8, 12, and 14 weeks post-injection. After the 14-week time point, mice were sacrificed for biochemical and pathological analysis.

A second experiment in Fabry mice was conducted to determine the effects of α-Gal A on Gb3 clearance. The following four vector doses of AAV8-FLT190 were assessed: 2 × 10^9^ vg/kg, 2 × 10^10^ vg/kg, 2 × 10^11^ vg/kg, and 2 × 10^12^ vg/kg. AAV8-FLT190 was injected into the tail vein of *Gla*-deficient (Fabry) mice 4 months of age. Plasma α-Gal A levels were measured at 7 to 7.5 months post-injection. Quantitative Gb3 levels in plasma and tissues were determined by tandem mass spectrometry (LC-MS/MS). Due to results showing differences in α-Gal A activity between males and females, only male mice were included in the analysis of the relationship between plasma α-Gal A and Gb3 clearance. α-Gal A biodistribution data and electron microscopy analysis was carried out for only the highest dose group (2 × 10^12^ vg/kg).

### NHP studies

Juvenile rhesus macaques were obtained from Shin Nippon Biomedical Laboratories (SNBL) USA Scientific Resource Center (Alice, TX) and housed at SNBL USA (Everett, WA). The following two studies were conducted: a 13-week GLP toxicology study in which NHPs received FLT190 at a dose level of 3 × 10^13^ vg/kg (3 males and 3 females), and a 26-week investigational study in which NHPs received FLT190 6 × 10^12^ vg/kg (3 males) or vehicle control (3 males). All study animals had been previously screened as negative for the presence of neutralizing antibodies (NAbs) against the AAVS3 vector. To assess the kinetics and durability of transgene expression, plasma was collected at various time points pre- and post-injection. Animals were followed for 13 weeks or 26 weeks before being sacrificed for biochemical and pathological analysis.

α-Gal A activity was measured at regular intervals in plasma and the level of h*GLA* mRNA in the liver was also assessed. Additional analyses included vector biodistribution, plasma anti-α-Gal A antibodies and plasma cTroponin levels.

### α-Gal A analysis

#### Plasma α-Gal A activity assay

α-Gal A activity was determined using the fluorogenic substrate 4-methylumbelliferyl-α-D-galactopyranoside as previously described [23]. The hydrolysis of the substrate 4-methylumbelliferyl-α-D-galactopyranoside to the products 4-methylumbelliferone and galactose by α-Gal A was stopped after 2 h incubation at 37 °C by the addition of alkaline buffer to inhibit further enzyme activity. α-Gal A activity was quantified by monitoring levels of the product, which fluoresces at a different wavelength to the substrate.

#### α-Gal A biodistribution

α-Gal A biodistribution or uptake of expressed α-Gal A into target tissues was determined by enzyme activity assays [23]. Briefly, tissues were cut into small pieces (approx. 300–500 mg) dissected on dry ice using a mortar and pestle and weighed to a total amount of ~0.3–0.5 g. Tissues were then homogenized with QIAGEN TissueRuptor for 30 s at the medium/high speed setting in 2 mL of lysis buffer (27 mM citric acid, 46 mM sodium phosphate dibasic pH 4.6, 1% Triton X-100, supplemented with 1x protease inhibitor cocktail tablet/10 mL [Complete Mini EDTA-free, Roche]) to avoid excessive foaming. Homogenates were then placed on ice for 10 min before being subjected to three rapid freeze-thaw cycles, followed by clarification by centrifugation at 10,000 *g* for 10 min. Supernatant was then collected and aliquoted to avoid repeated freeze-thaw, and subsequently stored at −80 °C until assayed. α-Gal A activity in different tissue supernatants were measured using the artificial substrate 4-methylumbelliferyl-α-D-galactopyranoside as described above. The total concentration of protein was measured in each sample using a bicinchoninic acid (BCA) BCA Protein Assay Kit (Pierce, Thermo Scientific) assay as per manufacturer’s protocol. This assay has a reported working range of 20–2000 μg/mL. Samples were diluted 1:5 and 1:10 and performed in duplicate. Total protein concentration was estimated using a 9-point BSA standard curve and was expressed as μg/mL. The ratio of α-Gal A activity to total protein concentration was then calculated and expressed as [nmol/hr/mL of α-Gal A activity]/[mg/mL of total protein].

#### mRNA analysis in liver

Total RNA was isolated from NHP liver using the Qiagen RNeasy Protect Mini Kit according to the manufacturer’s instructions. Approximately 30 mg fragments of frozen liver were placed into buffer RLT (supplied in the kit), homogenized with pellet pestles (Sigma-Aldrich) using a cordless motor (Kimble), followed by QIA shredder from Qiagen. The remainder of the purification procedure was carried out according to instructions.

RNA samples were assayed to measure vector-specific h*GLA* mRNA copy number. For cDNA synthesis, Invitrogen™ SuperScript™ IV VILO™ Master Mix was used according to manufacturer’s instruction. PCR amplification was carried out according to the manufacturer’s instructions. qPCR was performed on a QuantStudio™ instrument (Applied Biosystems) using PowerUp SYBR Green Master mix. Melting curve analysis was carried out at the end of cycling to confirm amplification of a single PCR product. All qPCR reactions were performed in duplicate and normalized to RLP13A mRNA levels.

PCR primers used in this study were the following:

h*GLA* Primer 1: qPCR-GLAco-Fw1: CCAACTACGTGCATAGCAAG

h*GLA* Primer 2: qPCR-GLAco-Re1: CGATTCGTTCCTGGTTGAAG product size: 399 bp

RLP13A primer 1-Fw1: CCTGGAGGAGAAGAGGAAAGAGA

RLP13A primer 2-Re1: TTGAGGACCTCTGTGTATTTGTCAA

Genome copy number from the tissues was calculated from the standard curve using a reference standard QPCR Master Stock (linearized FLT190 plasmid of known concentration).

### Statistical analysis

Data were analyzed using GraphPad Prism 7.03 (GraphPad Software, La Jolla, CA, USA). Data are presented as mean ± standard deviation (SD) and *p* < 0.05 is considered statistically significant. For in vitro studies, the difference between control and FLT190-treated samples in the supernatant activity and co-localization studies were analyzed using repeated measures analysis of variance (ANOVA) with Bonferroni post-test. The difference between control and treated samples in the uptake α-Gal A activity study was tested by unpaired *t*-test. For in vivo studies, two-way, repeated-measures ANOVA was performed to analyze α-Gal A expression after AAV8-FLT190/FLT190 or vehicle control over time (treatment x time factors). One-way ANOVAs with Bonferroni correction for multiple comparisons were conducted to compare the mean between all control and treatment groups. Student’s *t*-test was performed to compare levels of Gb3 or Lyso-Gb3 between treatment and control groups. The relationship between plasma α-Gal A to Gb3 was determined using a nonlinear fit regression model Marquardt method using SAS System.

The total number of animals was based on the minimum needed to properly characterize responses related to test article administration and thus to meet experimental objectives. The sample size calculation was in accordance with the 3 R principle and power calculation based on previous experimental data: https://eda.nc3rs.org.uk/experimental-design-group#PowerCalc.

Subjects were assigned to one of the treatment groups using block randomization; blind to the block size to avoid any potential selection bias.

## Results

### In vitro studies

#### GLA transgene expression and characterization in cultured hepatocytes

**Fig. 1:**
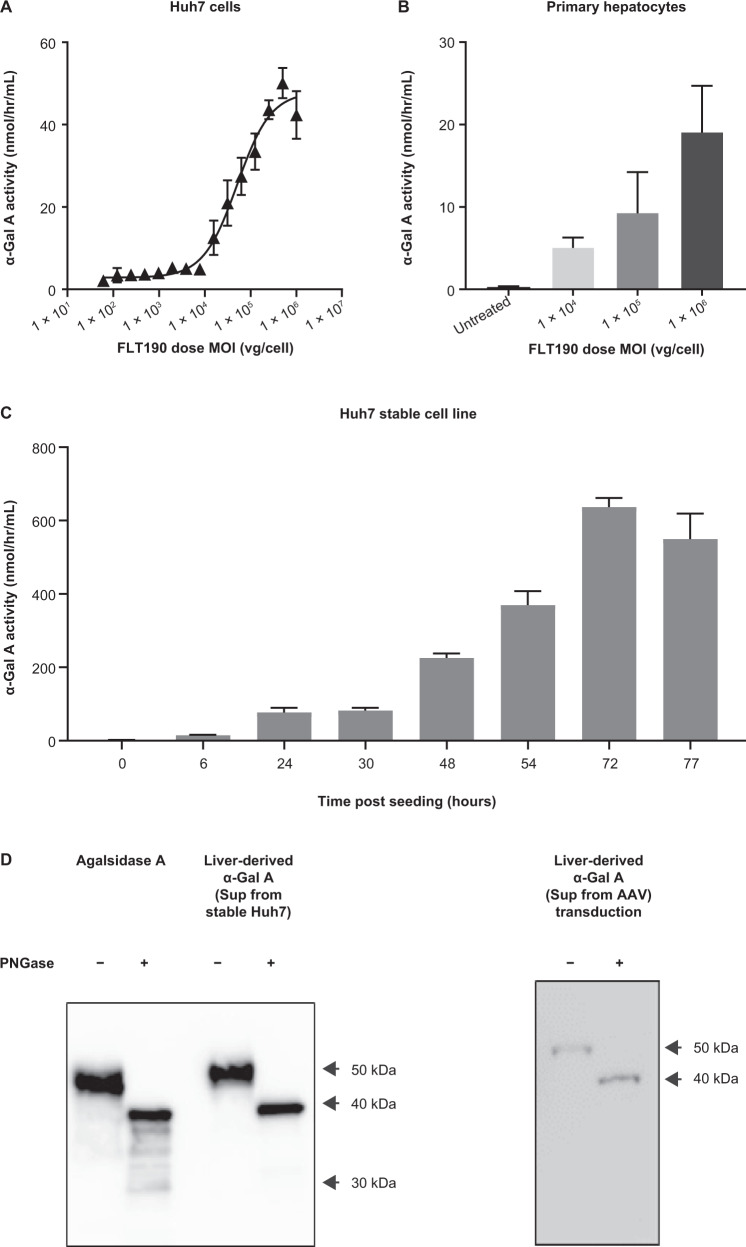
*GLA* transgene expression and characterization in cultured hepatocytes. Kinetics of α-Gal A secretion following FLT190 vector transduction in **A** Huh7 hepatocyte cell line and **B** human primary hepatocytes. **C** α-Gal A secretion from Huh7 cells engineered to overexpress α-Gal A. **D** Glycosylation analysis of α-Gal A produced from Huh7 cells following PNGase F digestion – representative Western blot analyses are shown. Agalsidase alfa was used as a comparator. Data are mean ± SD (*n* = 3). α-Gal A = alpha galactosidase A; AAV = adeno-associated virus; Huh7 = human hepatocyte cell line; MOI = multiplicity of infection; PNGase F = Peptide:N-Glycosidase F; SD = standard deviation; vg = vector genomes.

To evaluate transduction efficiency of FLT190 vectors in vitro, Huh7 cells (Fig. 1A) and human primary hepatocytes (Fig. 1B) were transduced at different MOIs (1 × 10^4^ vg/cell, 1 × 10^5^ vg/cell, and 1 × 10^6^ vg/cell) and the activity level of α-Gal A secreted in the media was assessed by an enzyme assay at 48 h post-transduction. For Huh7 cells, a 15-point dose-response curve (0 to 1 × 10^6^ vg/cell) was constructed. The effective concentration that gives 50% of the maximal response (EC50) was determined as a measure of the potency of vectors expressing the *GLA* transgene. A dose-dependent increase in α-Gal A secretion was observed in both cell types. Huh7 cells showed a non-linear dose-response in kinetics of α-Gal A expression with EC50 value of 5 × 10^4^ vg/cell and maximal activity level of 47 nmol/hr/mL. For human primary hepatocytes, three FLT190 MOIs were tested. A dose-dependent effect on the kinetics of α-Gal A expression was also observed, although expression was lower than observed in the Huh7 cell line.

A stable cell line, consisting of Huh7 cells engineered to overexpress α-Gal A from an integrated lentiviral vector (Lenti-CMV-eGFP-HPGK-GLAco), was also created as an additional α-Gal A producer cell line for secretion-uptake studies. The level of α-Gal A secretion was monitored from 0 to 77 h post-culturing (Fig. 1C), and a time-dependent increase in α-Gal A activity in the culture media was observed; activity levels at 24 h, 48 h, and 72 h were 77 nmol/hr/mL, 226 nmol/hr/mL, and 637 nmol/hr/mL, respectively.

The stable cell line has the same *GLA* codon-optimized transgene (GLAco) as the FLT190 expression cassette but driven by the CMV promotor. Regardless of the two delivery systems, the α-Gal A protein secreted from the stable cell line is expected to have the same glycosylation and maturation status as the AAV-transduced α-Gal A and will be a valuable tool for modelling in vitro uptake studies to characterize supraphysiological levels of exposure of the enzyme to the target cells and its efficiency on storage clearance.

The mature protein is comprised of two subunits of 398 amino acids (approximately 51 KD), each of which contains three N-linked glycosylation sites. To assess glycosylation status, supernatants of the culture media from the two expression systems were collected and treated with PNGase F before being analyzed by Western blot. As shown in Fig. 1D, the deglycosylation patterns and the shifting of bands were similar for both AAV- and lentiviral-vector produced α-Gal A and comparable to agalsidase alfa, which is currently in use as ERT in Fabry patients. These results confirmed that hepatic cell line-expressed α-Gal A was appropriately glycosylated similar to that produced by agalsidase alfa and can be used for uptake studies using Fabry-relevant cells.

### α-Gal A secretion in apical and basal layers

**Fig. 2:**
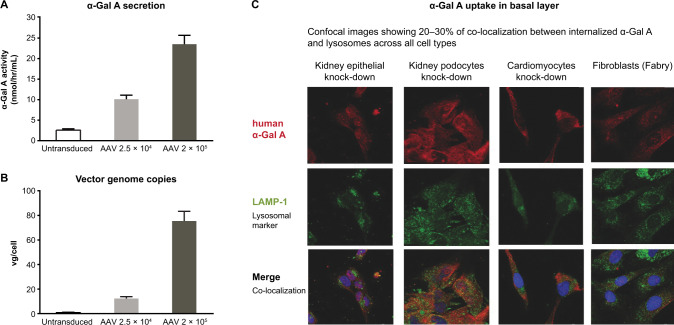
Uptake of α-Gal A by Fabry relevant target cells following transwell co-culture with FLT190 transduced Huh7 cells. **A** α-Gal A activity was measured in the culture media of basal transwells following co-culturing of AAV transduced Huh7 cells. **B** vg copy numbers. **C** Colocalization of internalized α-Gal A and lysosomes. Representative confocal images of co-cultured knock-down cell lines co-stained with HA-tag and LAMP-1 antibodies. α-Gal A = alpha galactosidase A; AAV, adeno-associated virus; HA-tag = hemagglutinin tag; Huh7 = human hepatocyte cell line; LAMP-1 = lysosomal-associated membrane protein 1; vg = vector genomes.

FLT190 transduction in cultured Huh7 cells in the apical layer of the AAV transwell system (Fig. S1) was examined at two different MOIs (2.5 × 10^4^ vg/cell, and 2 × 10^5^ vg/cell) at Day 3 post-transduction. As shown in Fig. 2A, FLT190 transduction of Huh7 liver cells cultured apically led to a dose-dependent increase in recombinant α-Gal A protein secretion into culture media. Measurement of vg/cell in the apical transwell layer following FLT190 transduction showed a dose-dependent increase in transduced cells that was consistent with the dose-dependent increase α-Gal A enzyme secretion (Fig. 2B). Finally, Q-PCR analyses were conducted to evaluate whether any AAV viral particles leaked across the membrane into the basal transwell during co-culturing, which could yield false-positive results. Results of these analyses showed that only apical transduced Huh7 cells contained a high level of vector genome copies. These data demonstrate that co-cultured cells in basal transwells did not contain AAV viral particles after co-culturing.

To demonstrate α-Gal A enzyme secretion in the AAV and stable co-culture systems, the level of enzyme activity in the basal culture media was measured across multiple co-culturing experiments. A dose-dependent increase in α-Gal A activity was observed 48 h following AAV transduction in all experiments, with an average of a 2-to-3-fold increase from baseline in secreted α-Gal A activity levels (Fig. 1A). The level of secreted α-Gal A in the stable cell line co-culturing system was also evaluated, and an approximate 12.5-fold increase from baseline was observed 48 h after co-culturing (Fig. 1C). These data demonstrate that AAV transduction of apical Huh7 cells, and/or seeding of stable cell line, led to an increase in basal secretion of α-Gal A enzyme, with the latter providing higher levels of enzyme production.

### Uptake of α-Gal A in key human cell lines

#### Confocal microscopy

The internalization of α-Gal A into lysosomal compartments was evaluated by confocal microscopy 48 h after co-culturing in both wild-type and knockdown cell lines of kidney epithelial cells, kidney podocytes, cardiomyocytes, and Fabry disease patient fibroblasts. The subcellular localization of α-Gal A was determined by differential immunofluorescent staining of HA-tag [24] and lysosomal associated membrane protein-1 (LAMP-1) following transduction with FLT190 at 3 different MOIs (0 vg/cell [untreated], 2.5 × 10^4^ vg/cell, and 2 × 10^5^ vg/cell).

Confocal microscopy confirmed intracellular distribution of α-Gal A in all cocultured cell lines as detected by anti-HA tag antibody (red channel), and lysosomes were visualized in the perinuclear region by immunodetection of LAMP-1 (green channel) (Fig. 2D). The merger of HA tag and LAMP-1 signals showed co-localization of α-Gal A and lysosomes across all cell types. These data provide evidence that all the cell lines tested were capable of internalizing into the cytoplasm α-Gal A expressed from hepatocytes, and a proportion of internalized enzymes was directed to the lysosomes (Fig. S2).

#### Western blot and densitometric analyses

To demonstrate uptake of α-Gal A in co-cultured cells, we measured total cellular levels of α-Gal A protein in key cell lines by Western blot and densitometric analysis following co-culturing with the AAV system. At 48 h post-co-culturing, a dose-dependent increase in total cellular α-Gal A protein normalized to GAPDH in wild-type and knockdown kidney epithelial, kidney podocytes and cardiomyocyte cell lines, as well as Fabry fibroblasts, was observed compared with an un-transduced control (Fig. S3). A direct relationship was observed between secreted enzyme levels and protein uptake in each cell line.

### Clearance of Gb3 storage from Fabry fibroblasts

Two different patient-derived Fabry fibroblast cell lines (81 and 107), patient-derived Fabry endothelial cells (IMFE1), and healthy (non-Fabry) fibroblasts were co-cultured with stable α-Gal A expressing Huh7 cells. Seventy-two hours post-co-culturing, densitometric analysis showed an increase in total cellular α-Gal A protein in these enzyme-deficient cell lines compared with untreated controls (Fig. S4A–C). The level of cellular α-Gal A protein in Fabry fibroblasts co-cultured with the stable cell line was found to be higher than those co-cultured with AAV-transduced Huh7 cells (20-fold increase versus 8-fold increase). This observation may be explained by the fact that Fabry fibroblasts were exposed to higher levels of α-Gal A via the stable cell line system (>150 nmol/hr/mL), as compared with AAV transduction (20 nmol/hr/mL), and further supports the finding that cellular exposure to higher levels of secreted enzymes leads to greater cellular uptake.

Tandem mass spectrometry was used to determine if uptake of transgene α-Gal A expressed from hepatocytes can functionally correct Gb3 lipid levels in cultured fibroblasts from Fabry patients. Gb3 lipid levels were measured in cell lysates of cultured fibroblasts exposed to α-Gal A following co-culturing as in Fig. S4A. As shown in Fig. S4D, the level of Gb3 in treated fibroblasts was normalized to a level similar to that observed in healthy controls. These data demonstrate that uptake of transgene-expressed α-Gal A can functionally correct excessive Gb3 accumulation in cultured Fabry fibroblasts.

### In vivo studies

#### Pharmacokinetics of AAV8-FLT190 in WT mice

**Fig. 3:**
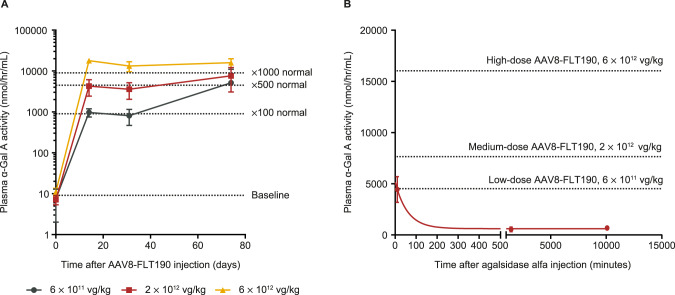
α-Gal A activity in plasma following administration of AAV8-FLT190 or ERT in wild-type (WT) mice. **A** Time course of α-Gal A enzyme activity levels in the plasma of WT mice that received AAV8-FLT190 6 x 10^11^, 2 × 10^12^ or 6 x 10^12^ vg/kg. Data are mean ± SD, *n* = 4–8 animals per time point. Plasma was collected by tail vein bleeding at the indicated times and the enzyme activity was measured using the fluorogenic substrate 4-methylumbelliferyl-α-D-galactopyranoside. **B** Plasma clearance curves after intravenous injection of agalsidase alfa at 0.2 mg/kg, in male WT mice. ERT pharmacokinetics using one-phase decay model data are mean ± SD, *n* = 5 animals per time point. Dotted line indicates plasma α-Gal A at steady state levels following AAV8-FLT190 in wild-type mice. α-Gal A = alpha galactosidase A; AAV8 = adeno-associated virus serotype 8; ERT = enzyme replacement therapy; SD = standard deviation; vg = vector genomes; WT = wild type.

AAVS3 was developed for increased human hepatocyte transduction, and is less efficient at transducing mouse hepatocytes; therefore, FLT190 genome (FRE1-*GLA*co) pseudotyped with an rAAV8 vector (AAV8-FLT190) was used for mouse studies. All AAV8-FLT190-treated animals exhibited a rapid dose-dependent increase in plasma α-Gal A activity by Week 2 and this high level of expression was maintained for the duration of the study (Fig. 3A, Table 1). The lowest dose of AAV8-FLT190 (6 × 10^11^ vg/kg) yielded an 830-fold increase in α-Gal A compared with the control group. Increasing the AAV8-FLT190 vector dose to 6 × 10^12^ vg/kg resulted in a marked elevation in plasma α-Gal A enzyme activity to 2500-fold greater than controls (Fig. 3A and Table 1). An analysis of area under the curve (AUC) in the AAV8-FLT190 dose groups showed a linear pharmacokinetic profile (R^2^ = 0.9901).

**Table 1:** Plasma α-Gal A enzyme activity in wild-type mice following treatment with AAV8-FLT190 or enzyme replacement therapy.

Treatment Group	C_max_ (nmol/hr/mL)	AUC_2week_ (U*hr/L)	Fold vs agalsidase alfa C_max_	Fold vs agalsidase alfa AUC	Fold vs agalsidase beta C_max_	Fold vs agalsidase beta AUC
Agalsidase alfa 0.2 mg/kg	4516	13,678	1	1	-	-
Agalsidase beta 1 mg/kg	67,932	62,111	-	-	1	1
AAV8-FLT190 6 x 10^11^ vg/kg	5134	103,501	1.14	7.6	0.08	1.7
AAV8-FLT190 2 × 10^12^ vg/kg	7648	154,183	1.69	11.3	0.11	2.5
AAV8-FLT190 6 × 10^12^ vg/kg	16,031	323,184	3.55	23.6	0.24	5.2

Comparing C_max_ of GLA enzyme activity levels in the plasma of C57BL6 wild type male mice administered at 6 × 10^11^, 2 x 10^12^ or 6 × 10^12^ vg/kg of FLT190 or ERT at 2 months of age. Table shows C_max_ and AUC comparison of 3 dose groups vs ERT and fold changes. AUC is expressed as U*hr/L where one unit is defined as the amount of enzyme required to hydrolyze 1 µmol/h of 4-methylumbelliferyl-α-D-galactopyranoside substrate at 37 °C.

α-*Gal A* alpha galactosidase A, *AAV*8 adeno-associated virus serotype 8, *AUC* area under the curve, *C*_max_ highest concentration of α-Gal A in plasma, *ERT* enzyme replacement therapy, *GLA* galactosidase, *vg* vector genomes.

An evaluation of the pharmacokinetic profiles of current ERTs (agalsidase alfa and agalsidase beta) was performed in parallel to AAV8-FLT190. Agalsidase alfa (0.2 mg/kg) or agalsidase beta (1 mg/kg) was administrated via tail vein injection to C57BL6 wild-type mice at 2 months of age (*n* = 15 per group). Mice were sacrificed at 10 min (*n* = 5), 24 h (*n* = 5) or 1 week (*n* = 5) post-injection for determination of plasma α-Gal A levels. Following administration of ERT, α-Gal A was cleared rapidly from the plasma. Assuming exponential decay, a modelled half-life of approximately 40 min was calculated with C_max_ assumed to be reached within 10 min following injection. The observed C_max_ and AUC of α-Gal A activity for agalsidase beta were greater than those of agalsidase alfa, which may be due to differences in dose (1 mg/kg and 0.2 mg/kg, respectively) (Table 1).

A comparison of the pharmacokinetic profiles of α-Gal A produced by AAV8-FLT190 with those produced by ERTs showed substantially greater α-Gal A enzyme exposure with AAV8-FLT190 than with both agalsidase beta and agalsidase alfa (Table 1). The α-Gal A expressed with the lowest dose of AAV8-FLT190 (6 × 10^11^ vg/kg) was comparable to the C_max_ of agalsidase alfa 0.2 mg/kg. At this level, greater AUC was observed for the AAV8-FLT190 dose group due to the steady state and stable α-Gal A levels in plasma (Fig. 3B).

### AAV8-FLT190 in *Gla*-deficient (Fabry) mice

#### Human α-Gal A expression

**Fig. 4:**
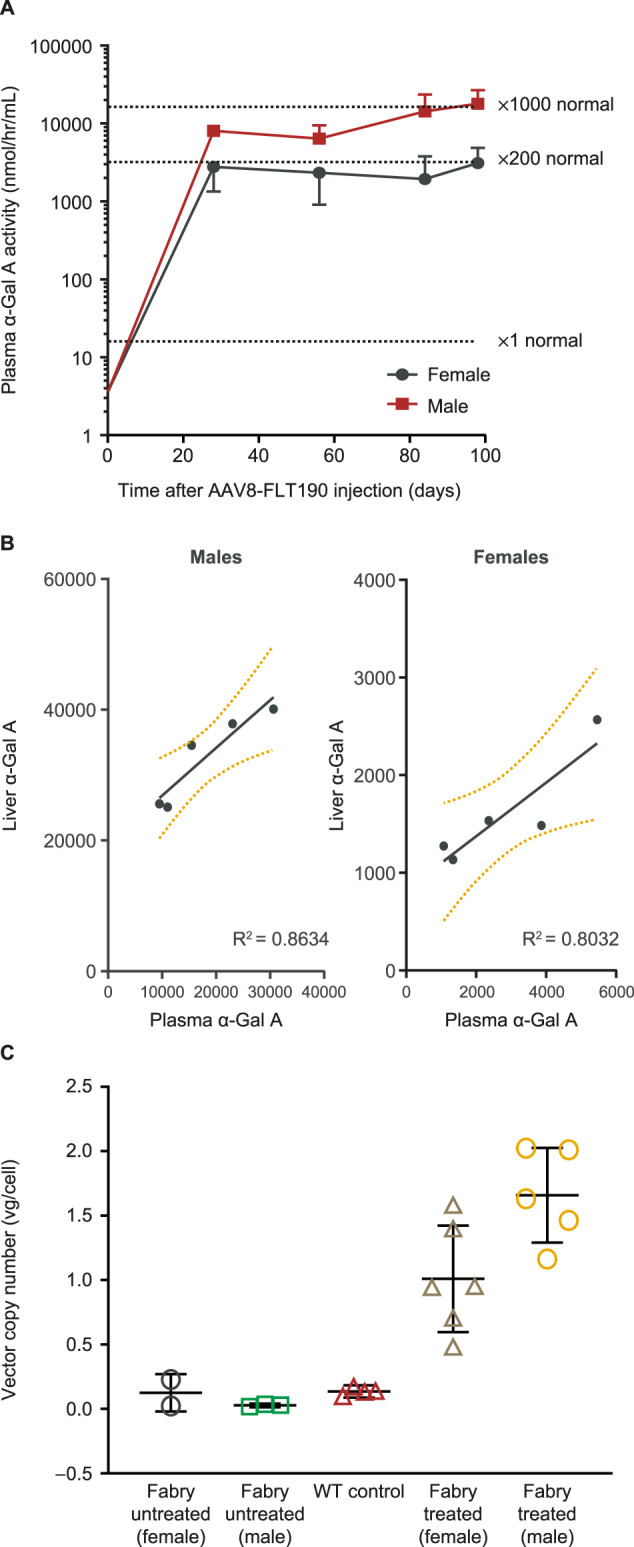
AAV8-FLT190 in *Gla*-deficient (Fabry) mice. **A** α-Gal A enzyme activity levels in plasma of *Gla*-deficient (Fabry) mice that received AAV8-FLT190 2 × 10^12^ vg/kg. Plasma was collected by tail vein bleeding at the times indicated and enzymatic activity was measured using the fluorogenic substrate 4-methylumbelliferyl-α-D-galactopyranoside. Data are expressed as mean ± SD, *n* = 5 to 6 animals per time point, males (*n* = 5) and females (*n* = 6). Wild-type α-Gal A level is shown as x1 normal, based on the measured activity from 4 animals. Data were analyzed using one-way ANOVA with Bonferroni correction for multiple comparisons. α-Gal A activity was significantly higher in AAV8-FLT190-treated mice when treatment group means were compared with untreated control means (*p* < 0.0001). **B** α-Gal A expression and secretion relationship for individual animals showing a positive correlation for liver homogenate to plasma α-Gal A enzyme activity levels. **C** Levels of vector genome present in murine liver lysate following administration of AAV8-FLT190 in Fabry mice. Amount of vector genome present in liver lysate was determined by QPCR using sets of primers targeting the FRE1 promoter. GAPDH was quantified using primers targeting mouse GAPDH. α-Gal A = alpha galactosidase A; AAV8 = adeno-associated virus serotype 8; FRE1 = Freeline-derived promoter; GAPDH = glyceraldehyde-3-phosphate dehydrogenase; QPCR = quantitative polymerase chain reaction; SD = standard deviation; vg = vector genomes; WT = wild type.

Fabry mice received AAV8-FLT190 via tail vein injection at 4 months of age; 2 × 10^9^ vg/kg (*n* = 10, 5 male and 5 female), 2 × 10^10^ vg/kg (*n* = 10, 3 male and 7 female), 2 × 10^11^ vg/kg (*n* = 10, 5 male and 5 female), and 2 × 10^12^ vg/kg (*n* = 11, 5 male and 6 female). Consistent with the results of studies in wild-type mice, Fabry mice treated with AAV8-FLT190 exhibited a rapid elevation in plasma α-Gal A activity levels, reaching peak levels by Week 4 post-treatment (Fig. 4A). This level of expression was maintained for the duration of the study (14 weeks) in all treated animals, with mean α-Gal A activity ranging from 4357 to 10,514 nmol/hr/mL. α-Gal A levels observed in male mice were significantly higher than in female mice (*p* < 0.002). Compared with untreated wild-type controls, the relative fold increases in α-Gal A activity levels in AAV8-FLT190 treated male and female Fabry mice were 1061 and 183, respectively, 14 weeks post-treatment. A strong positive correlation between α-Gal A enzyme activity in the liver and α-Gal A secretion to the plasma following AAV8-FLT190 administration was also demonstrated (Fig. 4B and Fig. 4C).

#### α-Gal A uptake and biodistribution

**Fig. 5:**
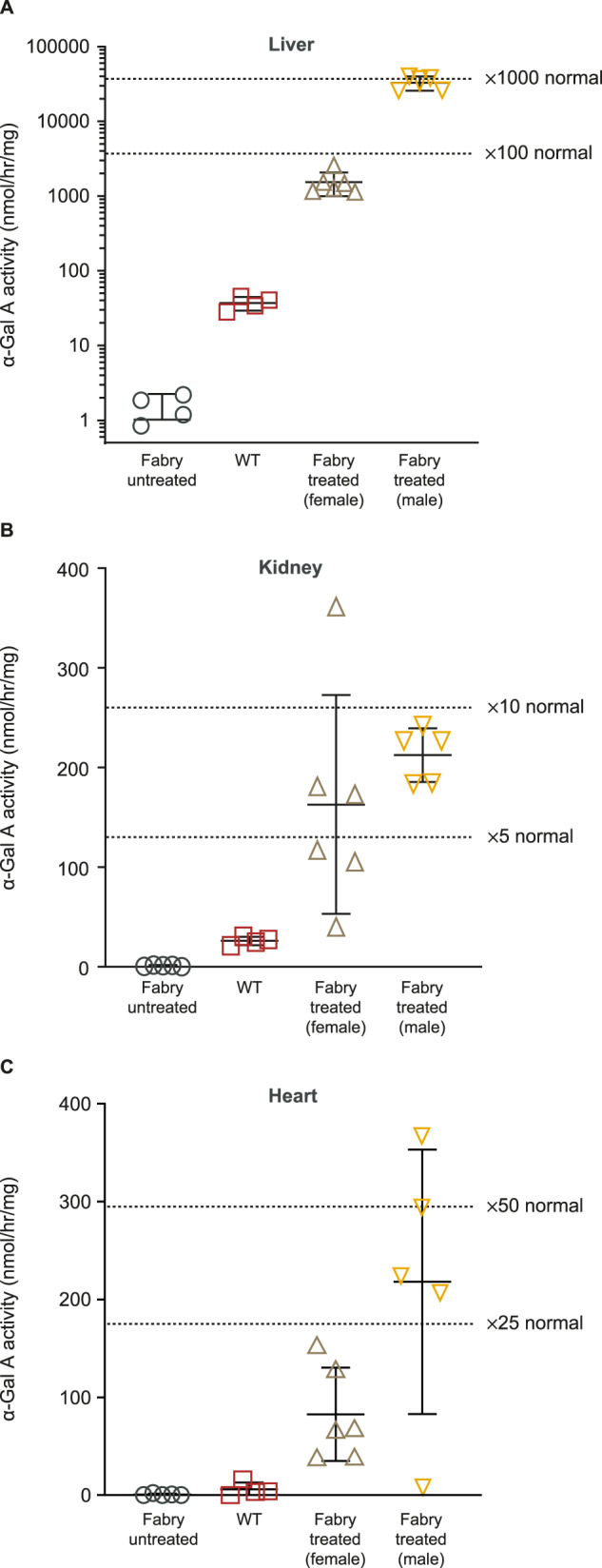
α-Gal A enzyme activity levels in the liver, kidney and heart of Fabry mice administered AAV8-FLT190 2 × 10^12^ vg/kg. α-Gal A enzyme activity was measured using the fluorogenic substrate 4-methylumbelliferyl-α-D-galactopyranoside. Data are mean ± SD, *n* = 4 to 6 animals per time point. One-way ANOVA with Bonferroni correction was used to compare mean of all treatment groups (AAV8-FLT190 treated vs. untreated (vehicle) controls, *p* < 0.0001). Fold increase was calculated based on WT untreated controls (*n* = 4). α-Gal A = alpha galactosidase A; AAV8 = adeno-associated virus serotype 8; SD = standard deviation; vg = vector genomes; WT = wild type.

α-Gal A enzyme activity was determined in tissue homogenates of liver, kidney, heart, spleen, and skin of Fabry mice at 14 weeks following administration of AAV8-FLT190. Tissue α-Gal A level was significantly elevated in the liver of AAV8-FLT190-treated mice, reflective of local production of the enzyme (Fig. 5A). Mean α-Gal A enzyme activity in the liver was 36.98 ± 7.31 nmol/hr/mg protein in WT mice and 1.25 nmol/hr/mg protein in Fabry mice. AAV8-FLT190 2 × 10^12^ vg/kg produced α-Gal A activity of 1527 nmol/hr/mg protein in female and 32,673 nmol/hr/mg protein in male Fabry mice, which were 41 times and 883 times greater, respectively, than observed in WT mice. These results demonstrated that the AAV8-FLT190 vector genome was efficiently expressed in liver.

Other tissues exhibited modest increases in α-Gal A activity compared with untreated mice, indicative of metabolic cross-correction and α-Gal A uptake (Fig. 5B, Fig. 5C, Fig. S5). With AAV8-FLT190, α-Gal A enzyme activity levels were 6- to 8-times greater in the kidneys and 14- to 37-times greater in the heart than the levels observed in WT mice. There was a strong positive correlation between plasma α-Gal A levels and tissue uptake, which varied across different tissues and organs.

#### Exposure and clearance of substrate

**Fig. 6:**
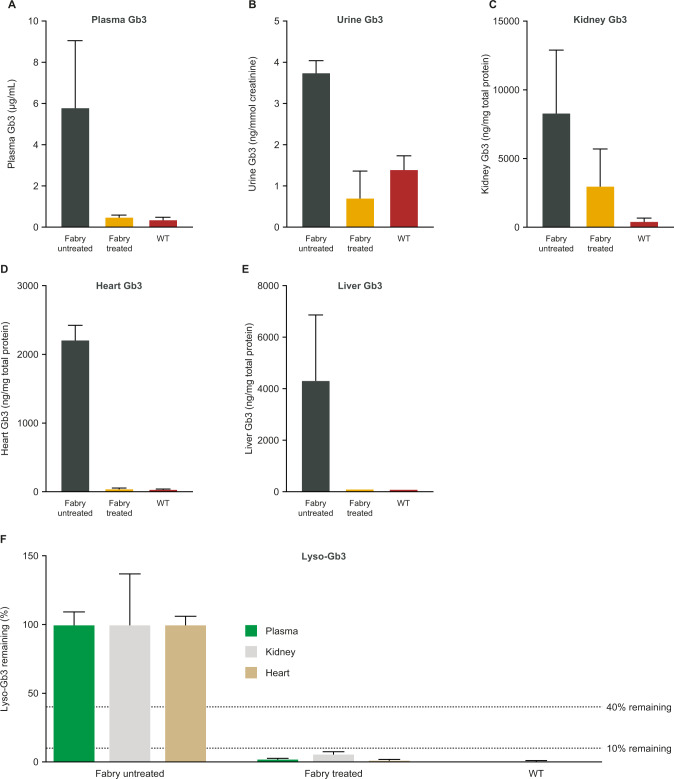
Levels of Gb3 and Lyso-Gb3 in plasma, urine and organs of Fabry mice administered AAV8-FLT190 2 × 10^12^ vg/kg. Data are mean ± SD, *n* = 4 animals per time point, analyzed 14 weeks after treatment at 7.5 months of age. *n* = 4 for untreated age-matched control group, *n* = 2 for WT mice. The relative reduction of Lyso-Gb3 is shown as % remaining storage. Unpaired *t*-test was used to compare means of all treatment groups. AAV8-FLT190 treated vs. untreated controls: plasma Gb3 (*p* = 0.0084); plasma Lyso-Gb3 (*p* < 0.0001); urine Gb3 (*p* = 0.02); kidney Gb3 (*p* = 0.045); kidney Lyso-Gb3 (*p* = 0.022); heart Gb3 (*p* < 0.0001); heart Lyso-Gb3 (*p* ≤ 0.0001); liver Gb3 (*p* = 0.0075). AAV8 = adeno-associated virus serotype 8; lyso-Gb3 = globotriaosylsphingosine; Gb3 = globotriaosylceramide; SD = standard deviation; vg = vector genomes; WT = wild type.

Sustained expression of α-Gal A resulted in significant reductions in levels of Gb3 and Lyso-Gb3 in plasma and organs relevant to Fabry disease (Fig. 6).

In liver (Fig. 6), Gb3 levels were similar to those observed in WT mice, whereas untreated control Fabry mice continued to accumulate Gb3 and Lyso-Gb3. In heart, the increase in α-Gal A activity following uptake completely normalized Gb3 and Lyso-Gb3 substrate levels to those observed in WT controls 14 weeks after treatment. In addition, plasma Gb3 and Lyso-Gb3 were practically eliminated 14 weeks after AAV8-FLT190 injection compared with the untreated age-matched controls. Gb3 levels in urine were also effectively normalized after injection of AAV8-FLT190.

Results are also expressed as residual Gb3 or Lyso-Gb3 content in the treated animals relative to age-matched untreated controls. Fourteen weeks after treatment, Gb3 levels were reduced by 91% in the plasma (*p* = 0.008), 64% in the kidney (*p* = 0.045), 98% in the heart (*p* < 0.0001), 97% in the spleen (*p* < 0.0001), and 99% in the liver (*p* = 0.0075) compared with untreated controls. Lyso-Gb3 levels were reduced by 98% in the plasma (*p* < 0.0001), 94% in the kidney (*p* = 0.022), and 98% in the heart (*p* < 0.0001) compared with untreated controls (Fig. 6F).

#### Minimal therapeutic α-Gal A prediction

**Fig. 7:**
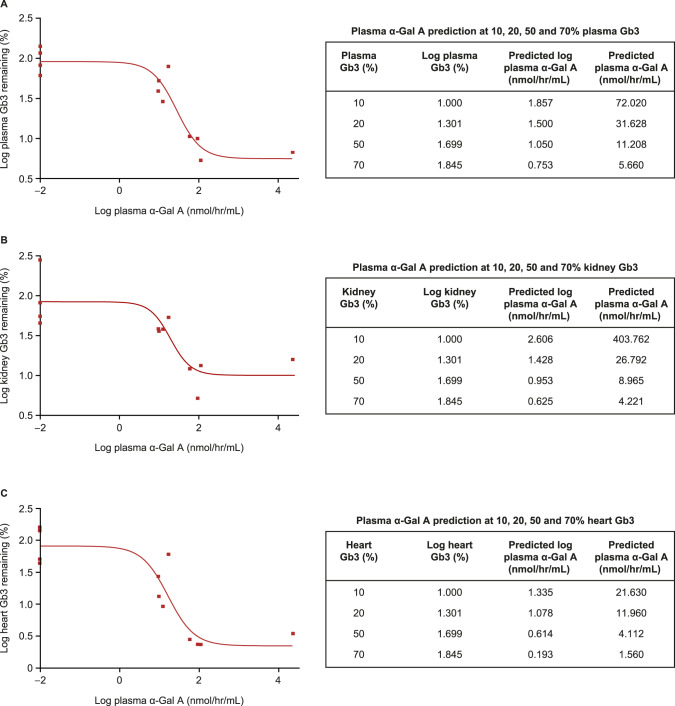
Correlating Plasma α-Gal A to Gb3. Correlating plasma α-Gal A to Gb3 using a nonlinear fit regression model Marquardt method in SAS System. Percentage (%) of remaining storage relative to untreated controls, Sigmoid (4PL) Fit: X = Log α-Gal A, Y = Logged Gb3 analysis is shown. The R^2^ values are the following: plasma = 0.89 (**A**), for kidney = 0.72 (**B**), and for heart = 0.82 (**C**). The main purpose of the curve fitting was to predict plasma α-Gal A values for various levels of Gb3 reduction (e.g. 50%), exploring log(plasma Gb3), log(kidney Gb3) and log(heart Gb3) values as the y responses. α-Gal A prediction at 10%, 20%, 50% and 70% remaining storage are shown in tables. Statistical packages SAS and GraphPad Prism were used for the analysis. α-Gal A = alpha galactosidase A; Gb3 = globotriaosylceramide.

α-Gal A-mediated Gb3 clearance was dose and time dependent. The range of plasma α-Gal A levels achieved in the AAV8-FLT190-treated mice varied from normal, to above normal (3 to 10-fold), to supra-physiological range (>1000 fold normal). Similarly, there was a wide range in the reductions in Gb3 storage in plasma and tissues. A positive correlation was observed between increase in plasma α-Gal A levels and a reduction in Gb3 storage in plasma, kidney, and heart (Fig. 7A‒C). The observed relationship that Gb3 reduction correlated with plasma α-Gal A activity enabled a prediction of the α-Gal A activity required to deliver a given level of efficacy, as defined by reduction in Gb3. α-Gal A vs. Gb3 analysis revealed a positive correlation between an increase in plasma α-Gal A levels and a reduction in Gb3 storage (R^2^ > 0.72). This approach allowed modelling of plasma α-Gal A required for specific levels of plasma/kidney/heart Gb3 clearance. Predicted α-Gal A for minimal efficacy (defined as 50% reduction in Gb3 storage in plasma/tissues) was within the normal physiological range, indicating the importance of continuous exposure to α-Gal A.

#### Pathological correction and durability of expression

**Fig. 8:**
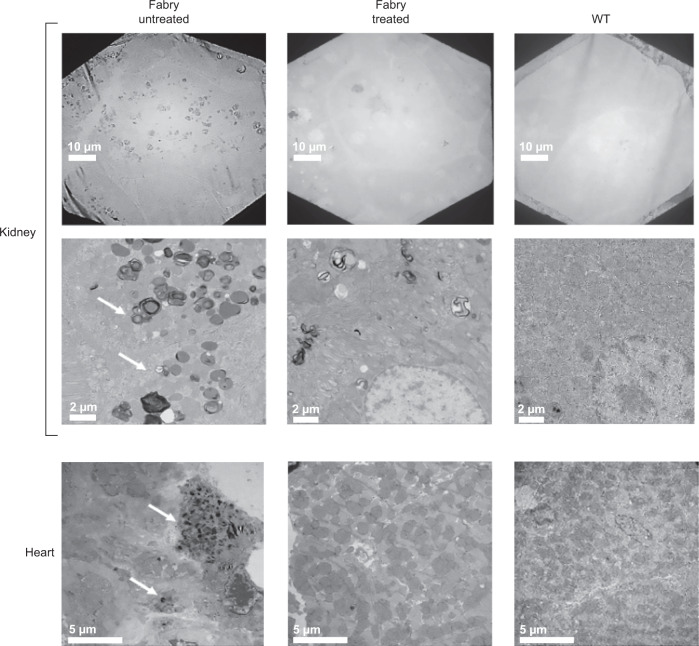
Representative electron microscopy images of kidney and heart tissues from Fabry mice untreated (left) and following administration of AAV8-FLT190 (right). Tissues from kidney, renal cortex and apex of the heart were processed for electron microscopy. The arrows indicate pathological changes resulting from Fabry disease-like features (storage inclusion bodies). Clearance of substrate was observed in AAV8-FLT190-treated mice compared with vehicle control Fabry mice. Representative pictures shown. The scale bar represents 10 µm and 2 µm for kidney, and 5 µm for heart. AAV8 = adeno-associated virus serotype 8; WT = wild type.

Storage of substrate in kidney and heart and endothelial abnormalities similar to those observed in Fabry patients previously have been described in the *Gla*-deficient mouse model [22]. We noted significant reductions in storage inclusion bodies in renal cell types and heart in AAV8-FLT190-treated animals (Fig. 8) with stable serum α-Gal A activity. Sustained, durable levels of plasma α-Gal A were demonstrated in Fabry mice up to 14 months of age after a single dose of an early development self-complementary (sc) codon optimized wild-type *GLA* proof-of-concept construct scAAV2/8-LP1-GLAco-SV40p 2 × 10^12^ to 2 x 10^13^ vg/kg (Fig. S6).

### NHP studies

**Fig. 9:**
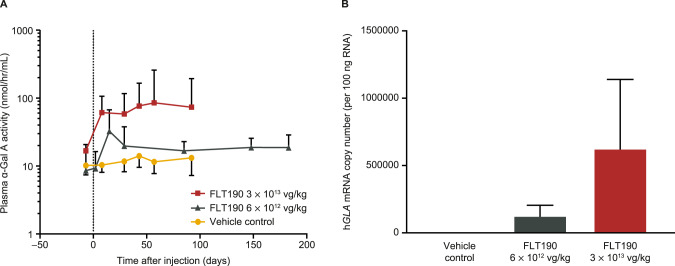
FLT190 evaluation in NHPs. **A** α-Gal A enzyme activity levels in the plasma of NHPs administered FLT190 3 × 10^13^ vg/kg or 6 × 10^12^ vg/kg dose. **B** h*GLA* mRNA levels in liver biopsies. Plasma was collected at the times indicated and the enzyme activity was measured using the fluorogenic substrate 4-methylumbelliferyl-α-D-galactopyranoside (nmol/hr/mL). Data are geometric mean ± SD, *n* = 6 animals per time point (vehicle control and 3 × 10^13^ vg/kg) or *n* = 3 animals per time point (6 × 10^12^ vg/kg). Two-way ANOVA with repeated measures was used to compare treatment groups. FLT190-treated vs. untreated (vehicle) control, *p* = 0.0003. α-Gal A = alpha galactosidase A; h*GLA* = human α-galactosidase A gene; mRNA = messenger ribonucleic acid; NHP = nonhuman primate; RNA = ribonucleic acid; SD = standard deviation; vg = vector genomes.

The pharmacokinetics of transgene expression following administration of FLT190 (pseudotyped with the AAVS3 vector) were investigated in rhesus macaques in the following two studies: a 13-week GLP toxicity study of a single dose of 3 x 10^13^ vg/kg that included 3 males and 3 females, and a 26-week investigational study of a single dose of 6 x 10^12^ vg/kg vector dose (3 males) and vehicle control (3 males and 3 females). Data on plasma α-Gal A activity and h*GLA* mRNA copy number from the two studies are combined and shown in Fig. 9A, B, respectively.

#### FLT190 α-Gal A expression

In the 13-week 3 x 10^13^ vg/kg NHP study, plasma α-Gal A activity increased rapidly from baseline within 1 week following FLT190 infusion and remained stable from Weeks 5 to 13 (Fig. 9A). Of the 36 plasma samples from FLT190-treated animals analyzed, mean α-Gal A specific activity at terminal stage was 115 nmol/hr/mL, with values ranging from 23 nmol/hr/mL to 294 nmol/hr/mL; baseline activity was between 14 and 21 nmol/hr/mL. On average, the relative increase in plasma α-Gal A activity was 5.5 times higher than normal in females and 7.6 times higher than normal in males 13 weeks post-treatment. A two-way ANOVA revealed significantly higher α-Gal A expression in the FLT190 group compared with the control group (*p* = 0.0003). In treated animals there was ongoing uptake and clearance of α-Gal A by the tissues, and an equilibrium state was maintained by continuous secretion of α-Gal A from transgene expression in the liver. Tissue α-Gal A activity was elevated in the liver of FLT190-treated NHPs, reflective of AAV-mediated expression of the enzyme. NHPs injected with FLT190 3 x 10^13^ vg/kg showed a mean α-Gal A level of 167 nmol/hr/mg protein as compared with 77 nmol/hr/mg protein in control NHPs, reflecting a 2.2-fold change between groups (data not shown). Consistent with plasma α-Gal A activity, high levels of h*GLA* mRNA were detected in the liver using RT-qPCR in the FLT190-treated animals compared with background levels detected in reference samples (Fig. 9B).

Two high-responder NHPs (#2003 and #2503) had α-Gal A levels in the liver that were 3.7- and 3.2-fold higher than normal levels (*p* < 0.0008), respectively. Other tissues in these two high-responder NHPs exhibited modest physiological elevations of α-Gal A levels compared with control NHPs, indicative of α-Gal A uptake into the target organs. For example, the α-Gal A levels in the kidney cortex reached 1.8 and 1.9 times above normal (*p* < 0.0001) in the two high-responder NHPs, whereas no significant uptake was observed in spleen and heart. In the FLT190 treated group, mean α-Gal A levels of 387 nmol/hr/mg protein were detected in the kidney cortex, 1.5 above that of the control group at 13 weeks post-dose. As for the high-responding animals, no significant uptake was observed in the spleen or heart.

AAV-mediated dorsal root ganglion degeneration and dorsal axonopathy of the spinal cord have been observed at the cervical, thoracic, and lumbar segments following the administration of AAV vectors in NHP [25]. The neuronal degeneration was predominantly noted when vectors were administered directly to the cerebrospinal fluid and when the dose was 1 x 10^13^ vg/kg or greater. Hence, we performed a histological examination of cervical, thoracic and lumbar dorsal root ganglion, and spinal cord tissues from this study. No neuronal degeneration was noted in any FLT190-treated NHPs.

In the 26-week NHP study, the pharmacokinetic profile of α-Gal A in plasma was evaluated in animals dosed with FLT190 6 x 10^12^ vg/kg (3 males) at 7 time points: once in acclimation (baseline), and at Weeks 1, 3, 5, 13, 22 and 26 post FLT190 infusion. The plasma α-Gal A activity data showed a rapid increase in α-Gal A levels from baseline to 2 weeks post AAV infusion, and that plasma levels remained stable from Week 5 to 26 (Fig. 9A). Of the 21 plasma samples analyzed from FLT190-treated animals, the average specific activity of α-Gal A at the end of the study (Day 183) was 19.8 nmol/hr/mL, with values ranging from 11 nmol/hr/mL to 24 nmol/hr/mL, whereas the baseline activity measured at pre-dose time point was between 3 and 17 nmol/hr/mL. After FLT190 treatment, physiological levels of α-Gal A reaching up to 57.2 nmol/hr/mL were observed in animal #1003. However, on average, the relative increase of plasma α-Gal A activity was 2 times normal at most time points except at Day 15 where 4 times normal peaked levels were observed.

#### Anti-α-Gal A antibodies in plasma

The levels of anti-α-Gal A antibodies in sera of NHPs dosed with FLT190 were determined by an ELISA using a rabbit anti-human α-Gal A monoclonal antibody as a reference standard positive control. Of the six 3 x 10^13^ vg/kg FLT190-treated NHPs, one male (#2003) exhibited a positive signal for anti-α-Gal A antibodies at Day 57 and Day 92. The other five NHPs remained negative for anti-α-Gal A antibodies; no specific antibodies against α-Gal A were detected at Day 29, 57, and 92 post-dose as compared with pre-dose samples. The anti-α-Gal A positive NHP #2003 had a titer of 40 and 320 at Days 57 and 92, respectively, and there was no evidence of neutralizing effects or drop in α-Gal A expression at the corresponding time points.

## Discussion

Studies were conducted to evaluate the preclinical characteristics of FLT190, an investigational AAV gene therapy in development as a potential treatment for patients with Fabry disease. FLT190 consists of a novel, potent, engineered capsid (AAVS3) containing an expression cassette with a codon optimized human *GLA* cDNA under the control of the liver-specific promoter FRE1. A liver-directed AAV gene therapy approach has the potential to provide an effective, one-time treatment for Fabry disease that would overcome the limitations of chronic frequent administration of ERT or PCT. Importantly, therapeutic efficacy of liver-directed gene therapy will only be achieved through efficient distribution of liver-derived α-Gal A among the multiple affected tissues to correct enzyme levels and reduce accumulated substrate.

We developed a co-culturing system to model liver-directed gene transfer for the treatment of Fabry disease in vitro. The model provided a dynamic environment to demonstrate *GLA* transgene expression, α-Gal A protein secretion, and subsequent uptake of secreted α-Gal A protein into key cell types affected by Fabry. AAV transduction of Huh7 cells, cultured apically, led to a dose-dependent increase in recombinant α-Gal A protein secretion into culture media and exposure of co-cultured cells to the recombinant enzyme for 48 h produced a concomitant dose-dependent increase in total cellular α-Gal A protein. Results of kinetic studies showed that the level of cellular enzyme uptake was sensitive to the level of secreted protein present in the culture media. These data demonstrated that exposure of key cell lines (both wild-type and *GLA*-knockdown) to trace amounts of α-Gal A protein led to effective internalization of the recombinant enzyme.

In all mouse studies, the FLT190 *GLA* transgene pseudo-typed with AAV8, rather than AAVS3, was administered because AAVS3 is not efficient at transducing murine hepatocytes. AAVS3 was specifically engineered to target human hepatocytes, and studies that compared *F9* transgene delivery using AAVS3 vs AAV8 showed minimal vector detection when transduced with AAVS3 in mice [17]. In vivo experiments showed that *GLA* can be expressed from the liver in a dose-dependent manner and that resulting α-Gal A enzyme can be secreted in an active form that is readily detected even in the context of normal background α-Gal A activity in wild-type mice. In *Gla*-deficient (Fabry) mice, a single injection of AAV8-FLT190 significantly increased α-Gal A activity – both in plasma and tissues affected in Fabry disease – and consequently decreased Gb3 accumulation in multiple tissues, compared with untreated controls. Gb3 clearance was also observed using electron microscopy, which revealed reduced storage inclusion bodies in the kidney and heart of AAV8-FLT190-treated Fabry mice. We also demonstrated durability of transgene expression in vivo over 26 weeks of follow up.

In wild-type mice, α-Gal A secretion from the liver after administration of AAV8-FLT190 was rapid with significant elevation of α-Gal A activity detected in the plasma as early as two weeks post-injection, and more importantly, supraphysiological expression was maintained throughout the course of the experiment. Following administration of ERT, α-Gal A was cleared rapidly from the plasma, with a half-life of approximately 40 min and C_max_ was reached within 10 min following injection. A comparison of the pharmacokinetic profiles of α-Gal A produced by AAV8-FLT190 with those produced by ERTs showed substantially greater α-Gal A enzyme exposure with AAV8-FLT190 than with either agalsidase beta or agalsidase alpha.

The efficacy of ERTs is limited with regard to their ability to affect some of the key organs involved in Fabry disease due to a short circulating half-life and/or variable uptake into different tissues [4]. The short half-life of ERT in the circulation and the every-other-week intravenous infusion cycle leads to a sawtooth pharmacokinetic profile with high levels immediately following infusion, which fall away to very low levels within days. Extensive depletion of storage and the observed correction of lysosomal disease pathology in these studies strongly support a gene therapy approach to Fabry disease; gene therapy may be superior to chronic biweekly intravenous infusions of ERT for sustained long-term supply of enzyme. Unlike ERT, the α-Gal A expressed from the transgene would be subject to patient-specific post-translational modifications (e.g., glycosylation patterns) that are important for uptake into key tissues [9] and may reduce the development of anti-drug antibodies.

We demonstrated that α-Gal A-mediated Gb3 clearance in Fabry mice with AAV8-FLT190 was dose and time dependent, and capable of reversing the pre-existing storage toward normal levels. The minimal and maximal efficacies were assessed by correlating α-Gal A activity to Gb3 reduction using a non-linear fit regression model Marquardt method. This approach allowed modelling of the plasma α-Gal A activity levels required for specific levels of Gb3 clearance in plasma, kidney, and heart. Data confirmed that even low levels of α-Gal A expression in plasma can lead to some clearance of Gb3 storage. Predicted α-Gal A for minimal efficacy (defined as 50% reduction in Gb3 storage in plasma/tissues) was within the normal physiological range, indicating the importance of continuous exposure to α-Gal A. Nevertheless, the level of α-Gal A expression achieved with this approach resulted in widespread reductions in Gb3 storage, indicating the potential for clinical benefits.

Administration of FLT190 (AAVS3 capsid with *GLA* transgene) to NHPs was also effective in producing increased α-Gal A activity compared with pre-treatment. These experiments indicated that stable bioactive human α-Gal A can be expressed from the liver of NHPs and that the resulting protein is secreted in an active form that can be readily detected even in the context of a normal background of α-Gal A activity originating from NHP *GLA*. Furthermore, α-Gal A secretion from the liver was rapid with elevations in α-Gal A activity being detected in the plasma as early as two weeks post-injection. The levels of α-Gal A were sustained throughout the course of the experiment, demonstrating the durability and stability of α-Gal A expression, and secretion for uptake by target organs. These results clearly demonstrated efficient expression of human α-Gal A from the FLT190 vector in the liver, a significant proportion of which is secreted into the blood and subsequently recaptured from the circulation by cellular uptake via mannose dependent and/or mannose independent mechanisms depending on cell type [26, 27].

Across our FLT190 mouse studies, higher levels of α-Gal A activity were observed in male compared with female mice. This finding is consistent with other reports in the literature in which gender differences in transduction efficiency and transgene expression have been observed across several different AAV serotypes [28–33]. For example, in the first study to investigate effects of gender on AAV gene transfer, Davidoff et al. (2003) found plasma levels of human factor IX were between 5- and 13-fold higher in male compared with female mice following gene transfer of an rAAV-2–based vector (rAAV-2 CAGG-FIX) encoding the human *F9* cDNA [28]. There is evidence that androgens are likely responsible for the enhanced transduction and expression after gene transfer in male mice; castration has been shown to reduce AAV transgene expression in males to levels observed in female mice [28], whereas transient exposure of female mice to testosterone prior to administration of AAV vector improved gene transfer to levels observed in male mice [28, 34]. Further research is needed to determine if gender differences in AAV transgene expression occur in humans. A study that evaluated supplementation of androgens in NHPs demonstrated that transgene expression in NHPs does not respond significantly to the supplementation with androgens [35]; these data suggest that the gender differences in AAV gene transfer to the liver may be specific to mice and unlikely to occur in humans.

While NHPs dosed with 3 x 10^13^ vg/kg FLT190 generated high levels of enzyme expression, low or no anti-α-Gal A antibodies were detected in the majority of NHPs. These results suggest that the use of the liver-specific promoter FRE1 was associated with a minimal host immune response to the encoded transgene product. This lack of a robust immune response to α-Gal A in five of six FLT190-treated NHPs likely accounted for the high and sustained expression levels of the enzyme observed.

Safety and tolerability of h*GLA* overexpression in NHPs has been reported previously with an AAV8-pseudotyped h*GLA* (dose of 2 × 10^13^ vg/kg), which demonstrated average α-Gal A plasma levels of 40 nmol/hr/mL [35, 36]. Plasma α-Gal A levels in NHPs treated with AAV8 vectors were >1.5 log lower than in mice [35, 36]. In our studies of AAV8-FLT190 in mice and the AAVS3 vector FLT190 in NHPs, the reduction in α-Gal A expression from mouse to NHP was similar; however, we observed that the actual levels of plasma α-Gal A in NHPs were at least five times greater than that with constructs using AAV8 in NHP [35, 36]. We believe these higher levels of plasma α-Gal A were achieved through a codon-optimized *GLA* gene, such that the codon sequence of each amino acid was modified to reflect sequences of proteins that were highly expressed in the human, the potent liver-specific promoter FRE1 and the AAVS3-pseudotyped vector, which has increased liver tropism [17]. In the first-in-human clinical trial (MARVEL-1), the starting dose of FLT190 was set as 7.5 x 10^11^ vg/kg. This dose was informed by the murine modelling of plasma α-Gal A activity levels required for Gb3 clearance and linear scaling to dose from NHP data to provide α-Gal A activity in plasma within the normal range (clinical assay normal range: 4.0–21.9  nmol/hr/mL).

There are four investigational AAV gene therapy products currently being evaluated as potential treatments for Fabry disease: Sangamo’s ST-920 product using AAV6 capsid carrying human *GLA* transgene [37], uniQure’s AMT-191 using AAV5 capsid carrying a *GLA* transgene [38], 4D Molecular Therapeutics’ 4D-310 using a cardiac-directed capsid (C102) carrying a *GLA* transgene under the control of a ubiquitous promoter [39], and our synthetic AAVS3 capsid with codon-optimized human *GLA* cDNA under the control of the liver-specific promoter FRE1 (FLT190). The success of these approaches is dependent on the durability of supraphysiological *GLA* transgene expression in the liver as well as the ability of liver-secreted α-Gal A to reach target organs to achieve optimal metabolic cross-correction of the affected tissues, with 4D-310 additionally seeking expression directly in heart and other tissues with a ubiquitous promoter.

## Conclusions

These studies demonstrate preclinical proof-of-concept of FLT190 for the treatment of Fabry disease. In vivo studies of FLT190 demonstrated several features of liver-directed gene transfer for Fabry disease that may have considerable therapeutic value, including the following: α-Gal A over-expression that drives cellular uptake; stable long-term α-Gal A expression; normalization of Lyso-Gb3 storage material in plasma and key disease tissues (kidney, heart); normalization of Gb3 storage material in plasma, heart, urine; and significant clearance of Gb3 storage material from kidney. The ongoing Phase 1/2 MARVEL-1 study of FLT190 in patients with Fabry disease (NCT04040049) will further elucidate the potential benefits of FLT190 gene therapy.

## Supplementary information

Supplementary materials

## Data Availability

The data that support the findings of this study are available from the corresponding author (Jey Jeyakumar) or Freeline Therapeutics (medinfo@freeline.life), upon reasonable request.
