# Supplementary material for: Preclinical evaluation of FLT190, a liver-directed AAV gene therapy for Fabry disease
Source: Gene Ther. 2023 Jan 11;30(6):487–502. doi: 10.1038/s41434-022-00381-y (PMC10284695; doi:10.1038/s41434-022-00381-y)

**Supplementary Information**

**METHODS**

***In Vitro* Studies**

***Co-culturing systems***

AAV transwell approach

*DAY 1: Cell seeding.* Two separate 24-well culture plates (designated as ‘Apical’ and ‘Basal’ plates) were prepared as shown in Fig. S1A.

Apical layer (α-Gal A producer cells) – Apical wells of a 24-well transwell plate (HTS Transwell 24-well permeable support with 0.4 um pore polycarbonate membrane and 6.5 mm inserts, Corning) were seeded with Huh7 (hepatocyte cell line) cells at a density of 5e4 cells/well, and basal wells were filled with 0.5 ml D-10 media only. Basal layer (target cells) - In a separate 24-well plate, the cell line of interest (human cardiomyocytes [AC16], human kidney epithelial cells [HK2], and human podocytes [AB8/13]) was seeded at 1x105 cells/well and this acted as the receiving basal wells. Poly-D-lysine coverslips were placed into corresponding wells for immunocytochemistry as required.

*DAY 2: AAV transduction.* Twenty-four hours post seeding, culture media was aspirated off from all apical and basal wells in the Apical transwell plate only (no work was required to be carried out on the Basal plate). All apical wells were then washed with 0.1 mL *ex vivo* media. Wash media was then removed from all wells and AAV at the desired MOI in *ex vivo* media were added to the apical wells in a final volume of 0.1-0.15 mL; 0.5 mL Ex vivo media was also added to the basal wells for cell maintenance. Five to six hours post-transduction, 0.1 mL D-10 was added to all transduced apical wells.

*DAY 3: Coculturing.* Apical plate – Culture media was removed from apical and basal wells (careful not to touch the transwell membrane in the apical well) in the Apical transwell plate. All wells were then washed with fresh media and replenished with 0.1-0.15 mL D-10. Basal plate – Culture media was removed from all wells and replenished with 0.5-0.6 mL fresh D-10. Apical transwells in the Apical plate were slowly lifted off and carefully placed into corresponding wells in the basal plate. The combined trans-well plates were incubated at 37^o^C for 48 h before analysis. For analysis, all basal cell monolayers in the Basal plate were washed briefly with 0.5M NaCl to remove any membrane bound protein.

Stable transwell approach

*DAY1: Cell seeding.* Two separate plates (designated plate ‘Apical’ and ‘Basal’) were prepared as shown in Fig. S1B. Apical plate – Apical wells of a 24-well transwell plate (HTS Transwell 24-well permeable support with 0.4 um pore polycarbonate membrane and 6.5 mm inserts, Corning) were seeded with Stable Huh7 cells at a density of 5x10^4^ cells/well, and basal wells were filled with 0.5 ml D-10 media only. Basal plate - In a separate 24-well plate, the cell line of interest was seeded at 1x10^5^ cells/well and this acted as the receiving basal wells. Poly-D-lysine coverslips were placed into corresponding wells for immunocytochemistry as required.

*Day2: Coculturing.* Apical plate – Culture media was removed from apical and basal wells (careful not to touch the transwell membrane in the apical well) in the Apical plate. All wells were then washed with fresh media and replenished with 0.15 mL D-10. Basal plate – Culture media was removed from all wells and replenished with 0.6 mL fresh D-10. Apical transwells in the Apical plate were then slowly lifted off and carefully placed into the corresponding wells in the basal plate. The combined transwell plates were incubated at 37^o^C for 72 h before analysis. For analysis, all basal cell monolayers were washed briefly with 0.5M NaCl to remove any membrane bound protein.

***Generation of knockdown (KD) cell lines by CRISPR-Cas9***

A panel of cell lines was generated to represent the key affected tissues in Fabry disease to demonstrate uptake of α-Gal A following co-culturing. These included human hepatocytes (Huh7), human cardiomyocytes (AC16), human kidney epithelial cells (HK2) and human podocytes (AB8/13). Furthermore, we used CRISPR RNA guided Cas9 nucleases to knockdown α-Gal A expression in these key cell lines to evaluate uptake in these enzyme-impaired cells as a model of Fabry disease. To test the effectiveness of α-Gal A sgRNA at triggering Cas9-mediated gene knockdown, cellular α-Gal A activity was measured in each knockdown cell line and compared with the wild-type cell lines.

***Generation of stable GLA Huh7 cell line***

The ABM® lentivirus packaging protocol was used to generate a stable Huh7 cell line expressing FLT190 (*GLA*co) from an integrated lentiviral vector (Lenti-CMV-eGFP-HPGK-GLAco). The titer of the lentivirus was determined using qPCR Lentivirus Titration Kit (ABM) according to manufacturer’s protocol. Huh7 cells were transduced with the above vector at MOI 10 in the presence of polybrene at a concentration of 4 µg/mL 6 times. Following infection, Huh7 cells were subjected to fluorescent-activated cell sorting (FACS) to identify the top 20% of EGFP-positive population. Infected Huh7 cells were then expanded and characterized using a GLA activity assay for stable expression.

***Western blot analysis***

For *in vitro* studies, protein extracts were isolated from cultured cells by passing through 21-gauge syringes in protein lysis buffer containing 62.5 mm Tris, 2% SDS, 10 mm dithiothreitol and 10 μL protease inhibitor cocktail/100 mL (Sigma Aldrich, Ireland). The homogenate was centrifuged at 8000 rpm. at 4°C for 20 minutes and the supernatant was stored at −80°C. Protein concentration was determined by BCA protein assay kit (Pierce, IL, USA) with bovine serum albumin (BSA) at 2 mg/mL as standards on 96-well plates according to the manufacturer's protocol. Approximately 30 µg of total protein was loaded in each lane. Protein samples were separated by electrophoresis on bolt 4-12% gradient SDS–polyacrylamide gels (Invitrogen) under reducing conditions and transferred to nitrocellulose membranes (Amersham Protran 0.45 µM, GE). Primary antibodies used were as follows: anti-galactosidase alpha (1:1000, ab168341 Abcam); anti-HA tag antibody (1:1000, ab137838 Abcam). Blots were washed with 1XTBS and incubated with horseradish peroxidase-conjugated (HRP-conjugated) polyclonal rabbit IgG secondary antibody (1:2000, Abcam). Anti-GAPDH antibody [6C5] (HRP) was used as loading control (1:2000, ab105428, Abcam). The blots were developed using enhanced chemiluminescent kit (Pierce Chemical Co.) and chemiluminescence signals were captured under continuous exposure using *Syngene G:Box chemi imaging system*.

Peptide-N-Glycosidase F, also known as PNGase F, is the most effective enzymatic method for removing almost all N-linked oligosaccharides from glycoproteins [Maley et al 1989]. For N-glycosylation analysis of GLA, aliquots of Huh7 culture supernatants were digested with PNGase F (New England Biolabs) using the manufacturer’s protocol.

***Immunocytochemistry (ICC)***

Cells grown on coverslips were fixed in 4% paraformaldehyde (PFA) at room temperature for 30 minutes and blocked in PBS containing 5% normal goat serum at room temperature for 30 minutes. Coverslips were then co-stained with anti-HA tag (1:200, ab137838, Abcam) and anti-Lamp-1 (1:200, ab25630, Abcam) antibodies at 4^o^C overnight in a humidity chamber. Coverslips were then washed three times in PBS for 5 minutes and incubated with secondary anti-rabbit IgG Cy3 (ab6939, Abcam) and anti-mouse IgG Cy2 (ab6944, Abcam) antibodies at room temperature for 1 hour. Following incubation, all coverslips were washed three times in PBS for 5 minutes and mounted onto glass slides with aqua-polymount (Polyscience) after nuclei-counterstaining with DAPI (1:5000). Stained cells were visualized using a confocal microscope (Nikon).

Images were processed with the program FIJI ImageJ (National Institutes of Health, Bethesda, MD; <http://rsb.info.nih.gov/ij/>). Quantitative analysis of colocalization was undertaken using the plugin ‘colocalisation threshold’ of ImageJ, which uses the threshold algorithm of Costes et al (2004). This yielded the single-channel specific Mander’s coefficient adjusted for threshold tM1. RGB.tiff images were subjected to background subtraction (separate colours) prior to channel splitting.

The colocalization of signals from two channels was evaluated using ImageJ software (US National Institute of Health, Bethesda, MD, USA) and the JACoP plugin. This plugin provides Pearson and Mander coefficient values. Both coefficients determine the degree of overlap between pixels from two different channels expressed in a range from 0 to 1, where 0 means no overlap (no correlation) and 1 represents total overlap (full correlation). The M1 coefficient estimates the degree of green signal overlapping red, and the M2 coefficient estimates the degree of red signal overlapping green.

***In Vivo* Studies**

***Electron Microscopy***

Kidney and heart tissue from treated and age-matched control mice (7.5 months of age) were prepared for electron microscopy. Small blocks of tissues were fixed with 2.5% glutaraldehyde and 4% paraformaldehyde in 0.1 M sodium cacodylate (SC) buffer pH 7.2. The fixed tissue was washed three times in 0.1 M SC buffer, postfixed with 1% OsO4 + 1.5% potassium ferrocyanide 0.1 SC in 4 °C cold room for 2 hours with vigorous shaking, washed in Milli-Q water (Millipore Corporation), incubated samples in 0.5% uranyl acetate overnight at 4 °C in the dark, washed and dehydrated through an alcohol series and processed for epoxy resin infiltration, processed for embedding and sectioning. Selected areas of the tissues were cut and processed for ultra-thin sectioning, stained with Reynold’s lead citrate, and the lowest magnification images (145x) were captured on a FEI Tecnai T12 transmission electron microscope (Tecnai) operated at 120 kV using a OneView® digital camera (Gatan, Inc.).

**RESULTS**

***In Vitro* Studies**

***Uptake of α-Gal A in key cell lines***

**Confocal microscopy.** To evaluate the level of colocalization between α-Gal A and lysosomes, confocal images of the red and green channels were utilized to calculate the threshold-adjusted Mander’s coefficient (tM1). In each cell line, three regions of interests (ROIs) were measured per image, and a total of 12 images per treatment was used to obtain an average threshold adjusted Mander’s coefficient. Using the threshold approach to evaluate pixel colocalization, the mean value for threshold-adjusted Mander’s coefficient (tM1) of red HA tag over green LAMP1 was found to be between 0.2-0.35 in all examined cell lines. This indicates that, in the threshold images, 20-35% of the red pixels colocalized with green pixels. Collectively, significant increases in tM1 were observed in FLT190-treated cells by more than 2-fold compared with untreated controls, but no statistical difference was observed between the two FLT190 MOIs (Fig. S2).

**Figure S1: Transwell co-culturing system approach.**

Schematic diagrams illustrating the workflow timeline of the A) AAV transwell approach and B) stable Huh7 transwell approach**.**

α-Gal A = alpha galactosidase A; AAV = adeno-associated virus; D10 = culture medium; Huh7 = human hepatocyte cell line.

**Figure S2. Quantitative analysis of lysosomal co-localization of GLA/LAMP-1 using threshold Mander’s coefficient (tM1)**

tM1 represent the fraction of co-localizing pixels from the overall number of pixels with intensity values above the given threshold calculated for the regions of interest (ROI) in an image channel; tM1 data mean ± SD were recorded using 12 confocal images (three ROI per image on average). Asterisks denote statistical significance determined by one-way ANOVA and Bonferroni comparison.

AAV = adeno-associated virus; GLA = galactosidase; Huh7 = human hepatocyte cell line; KD = knockdown; LAMP-1 = lysosomal-associated membrane protein 1; MOI = multiplicity of infection; SD = standard deviation; WT = wild-type.

**Figure S3.** **Western blot and densitometric analyses of α-Gal A protein uptake in multiple key cell lines.**

Western blot (top) and corresponding densitometric analysis (bottom) of α-Gal A protein uptake normalized to GAPDH in A) wild-type and knockdown HK2 kidney epithelial cells, B) wild-type and knockdown AB 8/13 podocytes C) wild-type and knockdown AC16 cardiomyocytes, and D) Fabry fibroblasts. Anti-galactosidase alpha antibody (1:1000, ab168341 Abcam) used to detect α-Gal A.

α-Gal A = alpha galactosidase A; AAV = adeno-associated virus; GAPDH = glyceraldehyde 3-phosphate dehydrogenase; Huh7 = human hepatocyte cell line; KD = knockdown; MOI = multiplicity of infection; WT = wild-type.

**Figure S4.** **Clearance of Gb3 storage in Fabry fibroblasts following uptake of α-Gal A from the cell culture media.**

A) Detection of cellular α-Gal A activity in non-Fabry fibroblasts and two different Fabry fibroblast cell lines (Fabry F81 and Fabry F107) and IMEF1 Fabry endothelial cell line 72 hours post co-culturing with stable Huh7 cells. B) Western blot detection of α-Gal A protein uptake in Fabry fibroblasts 72 hours post co-culturing with stable Huh7 cells. C) Western blot detection of α-Gal A protein uptake in Fabry endothelial cells 72 hours post co-culturing with stable Huh7 cells. D) Clearance of Gb3 storage in Fabry fibroblasts following uptake of α-Gal A from the cell culture media. Data are mean ± SD (n=3).

α-Gal A = alpha galactosidase A; Fabry F81 and Fabry F107 = Fabry fibroblast cell lines; GAPDH = glyceraldehyde 3-phosphate dehydrogenase; Gb3 = globotriaosylceramide; Huh7 = human hepatocyte cell line; IMEF1 = Fabry endothelial cell line; SD = standard deviation.

**Figure S5.** **Uptake of expressed secreted α-Gal A into the key target tissues**.

Plasma, kidney, and heart tissues were assessed at 10-months post vector administration to 1-month old mice and 11-months post AAV8-FLT190 administration to 3-month-old mice (2 x 10^12^ or 2 x 10^13^ vg/kg). Data suggest that the levels of α-Gal A antigen detected by Western Blot in kidney and heart (A) in contrast to the very low background levels of cDNA detected by RT-PCR (B) are clearly due to uptake of circulating enzyme from plasma and not due to *in-situ* expression from the vector genome in these tissues.

α-Gal A = alpha galactosidase A; AAV8 = adeno-associated virus serotype 8; GAPDH = glyceraldehyde 3-phosphate dehydrogenase; cDNA = complementary DNA; KO = knockout; RT-PCR = real-time polymerase chain reaction; vg = vector genomes; 1M = mice treated at 1 month and analyzed at 10 months post vector administration; 3M = mice treated at 3 months and analyzed at 11 months post vector administration.

**Figure S6. Evaluation of scAAV2/8-LP1-GLAco-SV40p vector in intermediate and late IV-injected groups of Fabry mice.**

Durability of plasma α-Gal A activity was measured at intervals post IV vector administration to A) Intermediate stage, 1-month old mice and B) late stage, 3-month old mice. Corresponding electron micrographs of Gb3 deposits in kidneys of 1-month and kidney and heart of 3-month old FD mice treated with low dose (2 x 10^12^ vg/kg) and high dose (2 x 10^13^ vg/kg) are represented. Note that intermediate stage mice were culled 10-months post-IV injection and late stage mice were culled at 11-months post injection. Age matched untreated Fabry mice were used as a control. Error bars represent mean ± SD (magnifications: x5000).

α-Gal A = alpha galactosidase A; FD = Fabry disease; Gb3 = globotriaosylceramide; IV = intravenous; SD = standard deviation; vg = vector genomes.

**Figure S1**

**
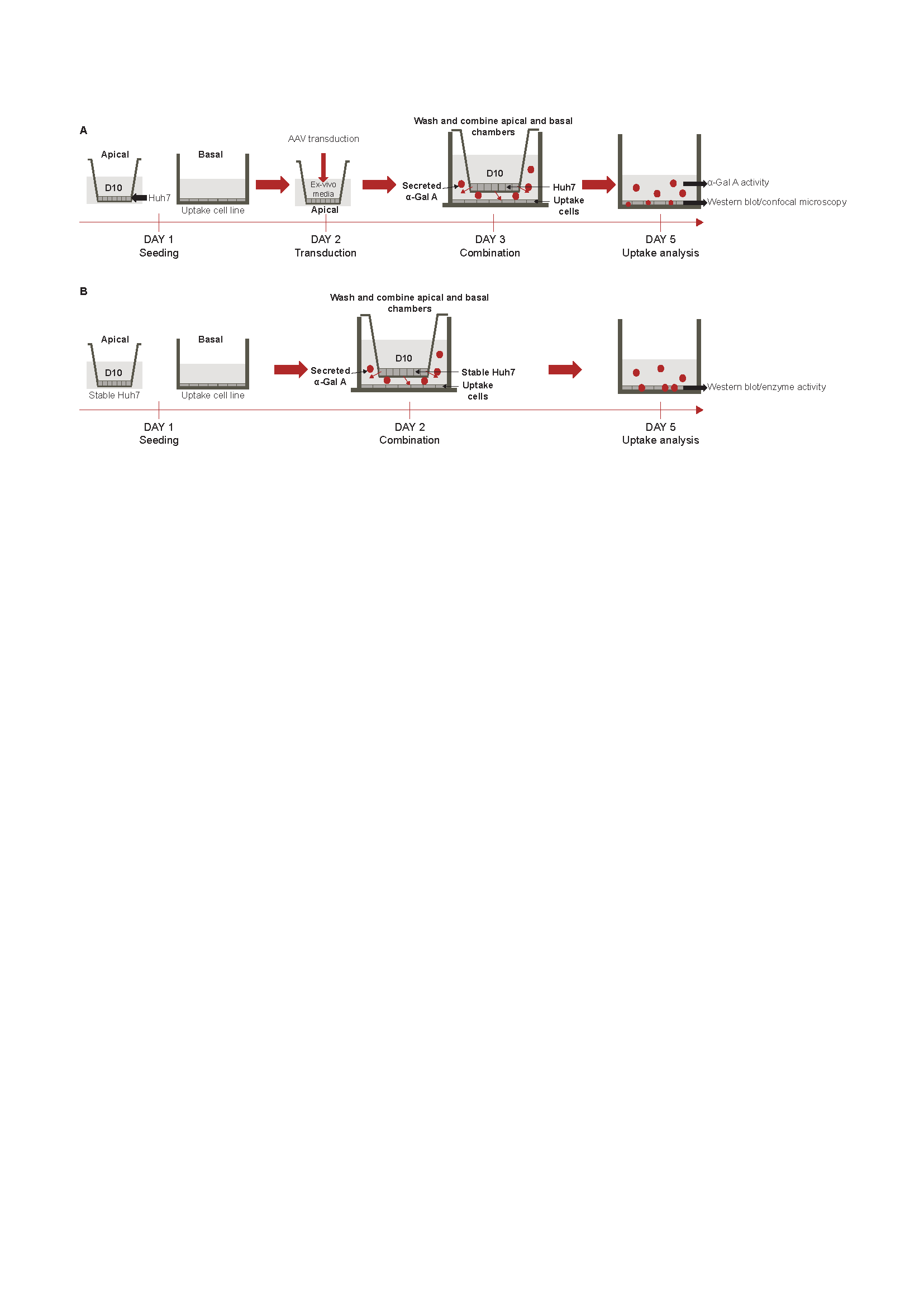
**

**Figure S2**

**
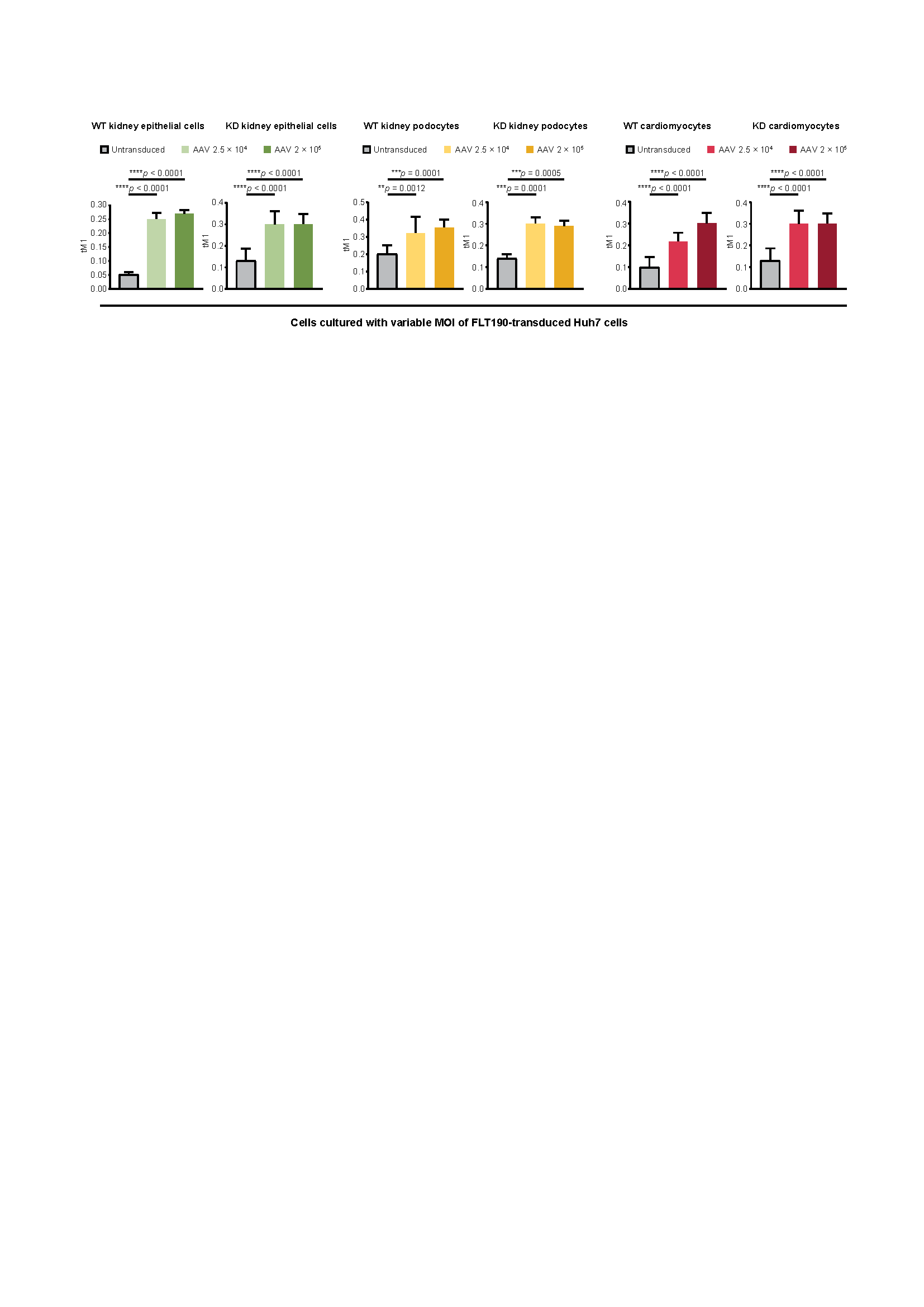
**

**Figure S3**

**
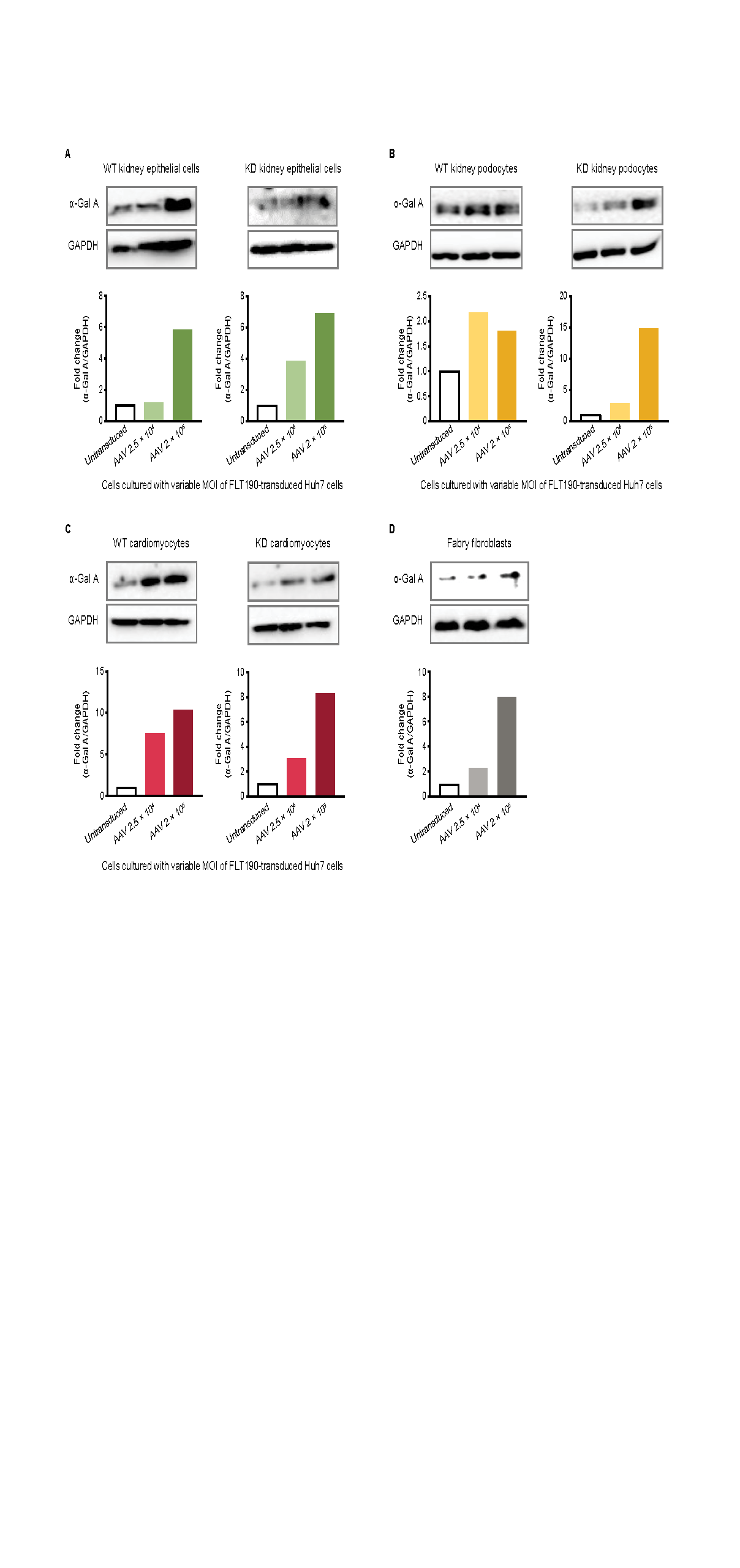
**

**Figure S4**


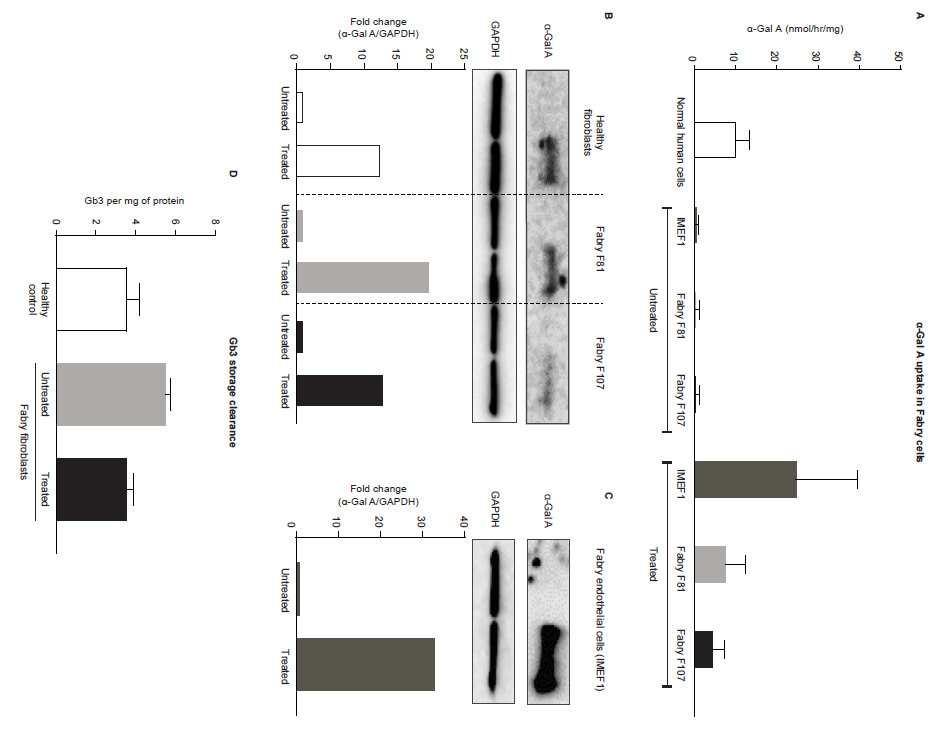


**Figure S5**

**
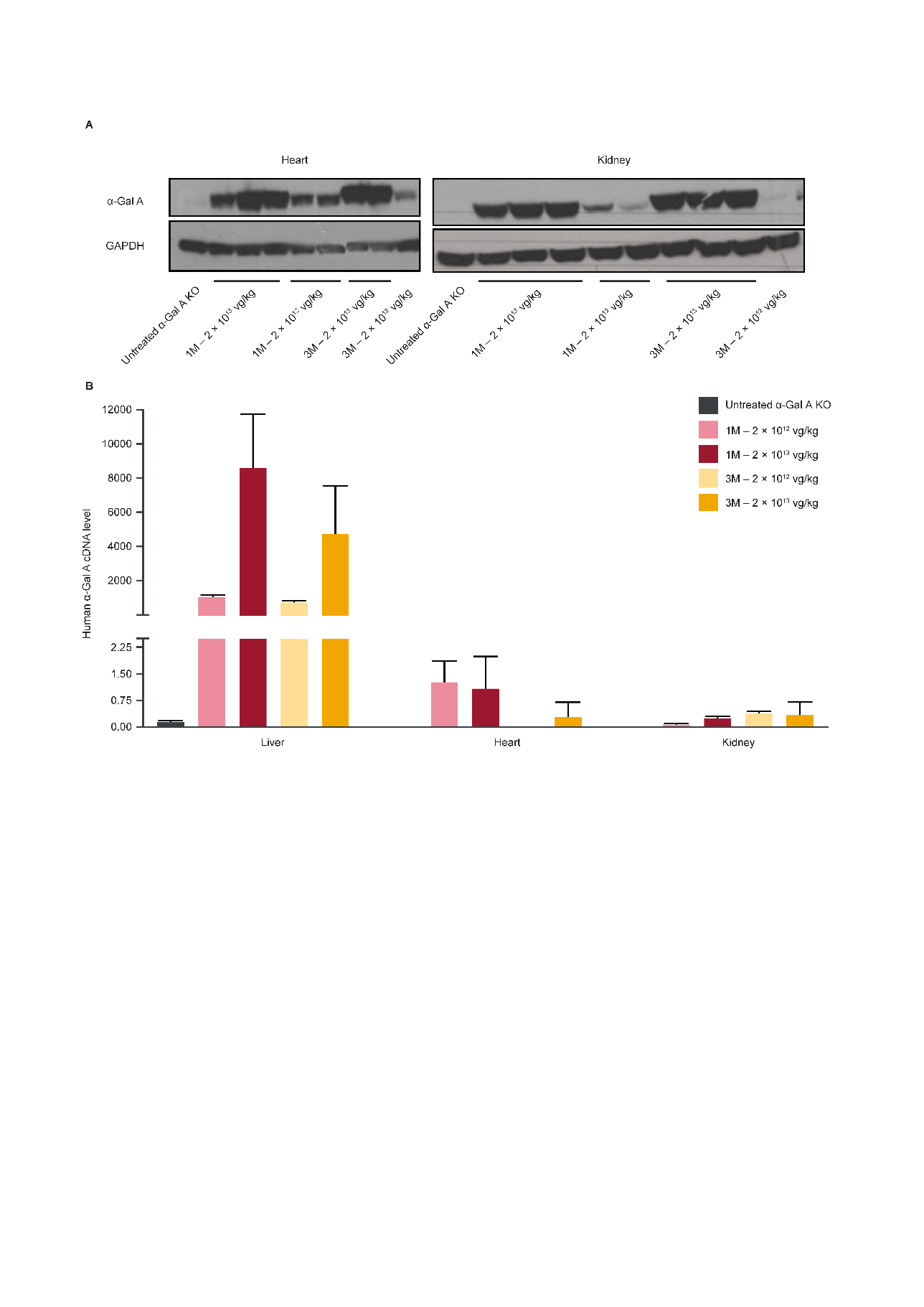
**

**Figure S6**


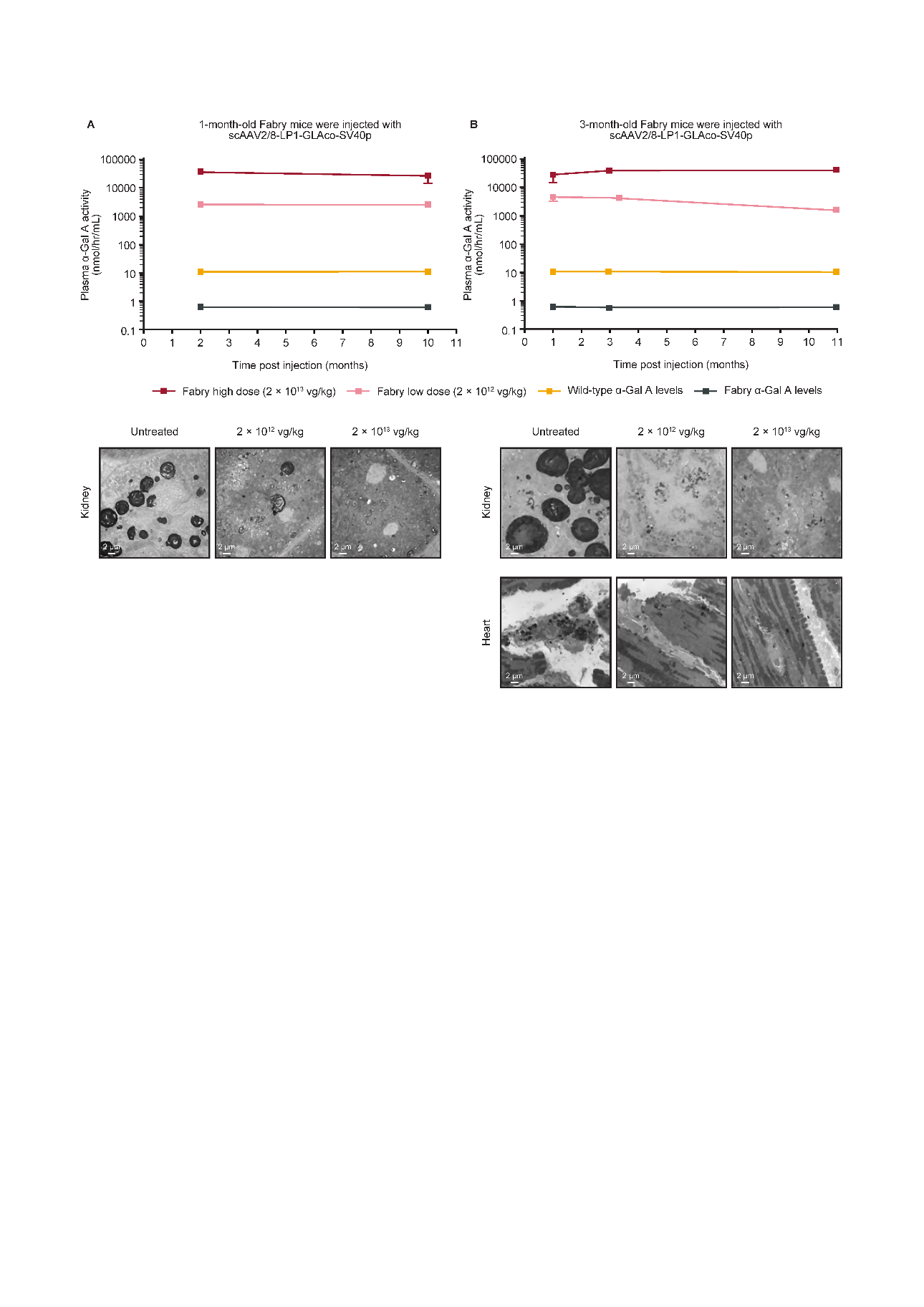

Supplement: Supplementary file 1 — Supplementary materials [file 41434_2022_381_MOESM1_ESM.docx]
